# Thiamin and Riboflavin in Human Milk: Effects of Lipid-Based Nutrient Supplementation and Stage of Lactation on Vitamer Secretion and Contributions to Total Vitamin Content

**DOI:** 10.1371/journal.pone.0149479

**Published:** 2016-02-17

**Authors:** Daniela Hampel, Setareh Shahab-Ferdows, Linda S. Adair, Margaret E. Bentley, Valerie L. Flax, Denise J. Jamieson, Sascha R. Ellington, Gerald Tegha, Charles S. Chasela, Debbie Kamwendo, Lindsay H. Allen

**Affiliations:** 1 USDA, ARS Western Human Nutrition Research Center, Davis, California, United States of America; 2 Department of Nutrition, University of California Davis, Davis, California, United States of America; 3 Gillings School of Global Public Health, University of North Carolina, Chapel Hill, North Carolina, United States of America; 4 Centers for Disease Control and Prevention, Atlanta, Georgia, United States of America; 5 UNC Project, Lilongwe, Malawi; 6 Division of Epidemiology and Biostatistics, School of Public Health, University of Witwatersrand, Johannesburg, South Africa; National Institutes of Health, UNITED STATES

## Abstract

While thiamin and riboflavin in breast milk have been analyzed for over 50 years, less attention has been given to the different forms of each vitamin. Thiamin-monophosphate (TMP) and free thiamin contribute to total thiamin content; flavin adenine-dinucleotide (FAD) and free riboflavin are the main contributors to total riboflavin. We analyzed milk collected at 2 (n = 258) or 6 (n = 104), and 24 weeks (n = 362) from HIV-infected Malawian mothers within the Breastfeeding, Antiretrovirals and Nutrition (BAN) study, randomly assigned at delivery to lipid-based nutrient supplements (LNS) or a control group, to investigate each vitamer’s contribution to total milk vitamin content and the effects of supplementation on the different thiamin and riboflavin vitamers at early and later stages of lactation, and obtain insight into the transport and distribution of these vitamers in human milk. Thiamin vitamers were derivatized into thiochrome-esters and analyzed by high-performance liquid-chromatography-fluorescence-detection (HPLC-FLD). Riboflavin and FAD were analyzed by ultra-performance liquid-chromatography-tandem-mass-spectrometry (ULPC-MS/MS). Thiamin-pyrophosphate (TPP), identified here for the first time in breast milk, contributed 1.9–4.5% to total thiamin. Free thiamin increased significantly from 2/6 to 24 weeks regardless of treatment indicating an active transport of this vitamer in milk. LNS significantly increased TMP and free thiamin only at 2 weeks compared to the control: median 170 versus 151μg/L (TMP), 13.3 versus 10.5μg/L (free thiamin, p<0.05 for both, suggesting an up-regulated active mechanism for TMP and free thiamin accumulation at early stages of lactation. Free riboflavin was consistently and significantly increased with LNS (range: 14.8–19.6μg/L (LNS) versus 5.0–7.4μg/L (control), p<0.001), shifting FAD:riboflavin relative amounts from 92–94:6–8% to 85:15%, indicating a preferred secretion of the free form into breast milk. The continuous presence of FAD in breast milk suggests an active transport and secretion system for this vitamer or possibly formation of this co-enymatic form in the mammary gland.

## Introduction

Thiamin (vitamin B1) and riboflavin (vitamin B2) are members of the B-vitamin complex involved in numerous biological processes, including carbohydrate, nucleic acid and amino acid metabolism [1–3]. During lactation, maternal nutrient requirements increase due to secretion of nutrients in breast milk to support growth and development of the infant [4], which can potentially lead to maternal depletion and/or infant deficiencies [5]. Severe thiamin deficiency causes infantile beri-beri [6–8]. More moderate depletion affects cardiovascular, muscular, nervous and gastrointestinal systems [9] and may be linked to sudden infant death syndrome [10]. Ariboflavinosis usually occurs along with other vitamin deficiencies causing growth retardation, anemia, degenerative changes in the nervous system, and impaired iron status [3, 9, 11, 12]. Even though vitamin deficiencies are mainly encountered in low income countries, an inadequate supply of thiamin and riboflavin to the infant through breast milk has also been described in well-nourished mothers. Böhm et al. reported that thiamin and riboflavin concentrations in breast milk of German mothers attained only 5–10% of the recommendations by the German Society of Nutrition (DGE) for newborns in the first two weeks (wk) postpartum [13], and breast milk from American mothers nursing their infants ≥ 2 wk had a median concentration of only 81% (range: 18 to 100%) of that assumed for setting the recommended Adequate Intake (AI) for infants [14].

The World Health Organization (WHO) recommends exclusive breastfeeding for the first 6 months of life [15–17] so adequate vitamin content of breast milk is crucial to infant health and development. All vitamers of a vitamin contribute to the total vitamin content. Thiamin in milk has been reported to be mainly present as thiamin-monophosphate (TMP) with some free thiamin [18, 19], while flavin adenine dinucleotide (FAD) represents the main riboflavin source complemented by free riboflavin and minute amounts of other flavin derivatives [20, 21]. However, little is known about the mechanisms surrounding the transport of these vitamins into breast milk or possible changes in vitamer uptake and their relative proportions due to supplementation.

Thiamin analysis is usually carried out using chromatographic separation and fluorescence detection after thiochrome derivatization, either after enzymatic digestion of the phosphorylated forms [22, 23] or direct conversion of free thiamin and its phosphate esters [14, 18, 19]. Riboflavin analyses have been conducted in a similar fashion based on its native fluorescent properties [11, 21]. While enzymatic pre-digestion will provide information about the total concentration of a vitamin, it does not provide information about its vitamers. Recently, we have reported a mass spectrometric method for analysis of free thiamin, free riboflavin, and FAD, enabling the simultaneous evaluation of the riboflavin vitamers [24]. Analyzing each vitamer separately allows the examination of the specific contribution of each form and monitoring of any possible changes in each vitamer throughout lactation and/or due to intervention such as supplementation or medication.

In this study we analyzed free and phosphorylated thiamin, and riboflavin and FAD in breast milk obtained from HIV-infected Malawian mothers within the Breastfeeding, Antiretrovirals and Nutrition (BAN) study, to investigate the contribution of each thiamin and riboflavin vitamer and the effect of lipid-based nutrient supplements (LNS) on the vitamer distribution at early and later stages of lactation.

## Materials and Methods

### Chemicals and Materials

Thiamin hydrochloride, thiamin-monophosphate, thiamin-pyrophosphate, riboflavin, flavin adenine dinucleotide, riboflavin-dioxopyrimidine-^13^C_4_,^15^N_2_, perchloric acid, phosphoric acid and sodium hydroxide were purchased from Sigma-Aldrich (St. Louis, MO). Methanol, acetonitrile, water (all LC-MS grade), phosphoric acid and potassium phosphate dibasic were obtained from Fisher Scientific (Fair Lawn, NJ). Potassium ferricyanide (III) was purchased from Acros Organics (Geel, Belgium); caffeine-trimethyl-^13^C_3_ from Cambridge Isotope Laboratories (Andover, MA) and ammonium formate from Hampton Research (Aliso Viejo, CA). HPLC screw-cap amber vials and inserts were obtained from Supelco (Bellefonte, PA). LC-vial caps (PTFE/silicone) were purchased from Waters (Milford, MA) and Agilent Technologies (Santa Clara, CA), and ultrafree centrifugal filters Durapore® PVDF 0.1μm from Millipore (Billerica, MA).

### Human Milk Samples

Existing human milk samples from the BAN study in Lilongwe, Malawi, were used for analysis. Details of the BAN study are described elsewhere [25–27]. For the purpose of this analysis, only milk samples from women receiving LNS (n = 185) and the control group (n = 177) were used. The effects of HIV treatment and LNS on milk B-vitamins have been discussed elsewhere [28]. The intervention started within 36 hours of delivery and continued to 28 wk postpartum. At delivery, all participants, regardless of the assigned treatment group, and their newborn infant underwent a 7-day perinatal ARV regimen [25]. No further ARV treatment was provided to these two study groups. Two sachets of LNS (70 g each, Nutriset, France; www.nutriset.fr) were given to the participants in the LNS group for daily consumption, providing 746 kcal/d and the Recommended Dietary Allowance (RDA) during lactation (**Table 1**). Breast milk samples were collected at weeks 2 or 6 and 24 during regular study visits and frozen instantly; the 6 wk samples (LNS: n = 45; control: n = 59) were collected only when 2 wk samples (LNS: n = 140; control: n = 118) were not available. Patients records were anonymized and de-identified prior to analysis. Samples were shipped on dry ice to the Centers for Disease Control and Prevention in Atlanta and stored at -70°C until analyzed at the USDA, ARS Western Human Nutrition Research Center (WHNRC) in Davis, CA.

**Table 1:** LNS composition formulated for lactating women and relative amount of micronutrients in LNS (x-fold) compared to the RDA.

Nutrient	Amount per 2 packets of LNS^1^ (140g)	x-fold of RDA for lactating women 19-30y
Energy	746 kcal (3120 kJ)	
Protein, *g*	20.8	
Iron, *mg*	15	1.7
Zinc, *mg*	19	1.5
Phosphorus, *mg*	1200	1.7
Selenium, μ*g*	75	1.1
Thiamin (B1), *mg*	1.6	1.1
Riboflavin (B2), *mg*	1.8	1.1
Niacin, *mg equiv*	20	1.2
Pyridoxine (B6), *mg*	2.2	1.1
Cyanocobalamin (B12), μ*g*	2.6	0.9
Ascorbic acid (C), *mg*	100	0.8
Alpha-Tocopherol (E) *mg*	12	0.6
Folic acid, μ*g*	300	0.6
Iodine, μ*g*	200	0.7
Potassium, *g*	1.1	0.2
Magnesium, *mg*	124	0.4
Copper, *mg*	0.3	0.2
Calcium, *mg*	294	0.3

^1^Ingredients: ground peanuts, dried skimmed milk, vegetable fat, sugar, multivitamin-mineral premix; Nutriset, France (www.nutriset.fr).

LNS, lipid-based nutrient supplement; RDA, Recommended Dietary Allowance (from Institute of Medicine [29]).

Informed consent was obtained from all mothers. This research was approved by the Malawi National Health Science Research Commission, the Institutional Review Board at the University of North Carolina Chapel Hill, the U.S. Centers for Disease Control and Prevention, and the Institutional Review Board of the University of California, Davis (Clinical Trials.gov #NCT00164762).

Pooled breast milk, provided by an apparently healthy donor from the Sacramento, CA area, was used for method development and validation and for quality control purposes.

### Biochemical Analyses

Sample preparation was carried out on ice under subdued light. Free riboflavin and FAD were analyzed by UPLC-MS/MS as previously described in detail [24]. Briefly, samples were subjected to protein precipitation and non-polar constituents were removed by liquid-liquid extraction prior to analysis. Quantitation was carried out by ratio response to an internal standard.

Free thiamin, thiamin-monophosphate (TMP) and thiamin-pyrophosphate (TPP) were analyzed using HPLC-fluorescence detection of their thiochrome derivatives after pre-column derivatization modified from Stuetz et al. [18, 19]. Pre-chilled perchloric acid (20 μL, ~70%) was added to 250 μL milk and mixed vigorously for 1 min before centrifugation for 10 min at 5°C (14000 rpm, SORVALL® LEGEND RT refrigerated benchtop centrifuge, Asheville, NC). The supernatant (200 μL) was transferred into a fresh 1.5mL amber microcentrifuge tube and 70 μL of freshly prepared potassium ferricyanide (12 mM) solution in sodium hydroxide (NaOH, 3.3 M) was added to start the thiochrome reaction. The samples were mixed briefly before quenching the reaction by adding 25 μL of 1M phosphoric acid (H_3_PO_4_). The neutralized samples were filtered and analyzed. Pooled breast milk from one apparently healthy donor was used as a control sample and prepared with every set of analyses (inter-assay variation of the pooled milk sample for all thiamin vitamers: 8.0–8.2% over 8 weeks, n = 80). Quantification was carried out using a 7-point external standard curve. Recovery was determined by standard addition experiments over 8 weeks: TPP 101.9% ± 6.3, TMP 118.7% ± 14.5, and free thiamin 121.7% ± 10.7. Recoveries >100% are most likely due to differences in standard curve and sample matrices. The analysis was carried out using an Agilent 1200 HPLC System equipped with a fluorescence detector (l_ex_: 367 nm, l_em_: 435 nm) and operated by ChemStation Rev. B.02.01.SR1 (Agilent Technologies, Santa Clara, CA). The samples were kept at 8°C in the autosampler and 30 μL was injected onto an Agilent Eclipse Plus C18 (4.6 x 150 mm, 5 mm) column protected by a SecurityGuard C18 (4 x 3.0 mm) guard column (Phenomenex, Torrance, CA) at 40°C. 150 mM potassium phosphate dibasic (aqueous, pH 7.0, solvent A) and methanol (solvent B) served as mobile phase at a flow rate of 1.5 mL/min and an 8 min gradient as follows: 0 min (85% A), 1 min (80% A), 3 min (80% A), 6 min (50% A), 7 min (85%), 8 min (85% A).

### Statistical Methods

Results were analyzed as concentrations of free thiamin, TMP, TPP, total thiamin, free riboflavin, FAD, and total riboflavin in milk. Participants were separated into two sub-groups based on their initial value being at either 2 or 6 wk postpartum (2 wk sub-set = 2 and 24 wk, 6 wk sub-set = 6 and 24 wk) and by treatment (LNS or control group). The distributions of concentrations were assessed for normality and transformations performed to normalize the variables: logarithmic transformations were performed on TPP, free thiamin, total thiamin, free riboflavin, and total riboflavin, while FAD was subjected to square root transformation. TMP had a normal distribution and was not transformed. Means were compared by paired t-test to evaluate changes over time within treatment group (2 to 24 wk; 6 to 24 wk); for evaluation of treatment effects at the time points of sample collection (2 or 6 wk, 24 wk) means were compared by t-test. P-values < 0.05 were considered to be statistically significant. SAS® statistical software 9.3 (SAS Institute, Cary, NC) was used for all statistical analyses.

## Results

### Maternal characteristics at initial time point

Maternal characteristics revealed no significant differences within and between the 2 wk- and 6 wk sub-groups (**Table 2**). The average BMI was within normal range and mothers self-reported a 92% compliance of LNS consumption, based on adherence reports collected over 5 follow-up visits. The self reported frequency of EBF was 96% at 21 wk portpartum [25, 30].

**Table 2:** Characteristics of participants in the Breastfeeding, Antiretrovirals, and Nutrition (BAN) study at the initial time point (no significant differences within and between subgroups).

Characteristic	Sub-group	Control			LNS^1^		
		n	median	IQR	n	median	IQR
Age, y	2 wk	117	25	(22–29)	139	26	(22–30)
	6 wk	59	25	(23–30)	44	25	(22–30)
Postprimary education, %	2 wk	118	33.9		140	37.1	
	6 wk	59	42.4		45	40.0	
Literacy, %	2 wk	113	76.1		136	77.2	
	6 wk	59	83.1		43	69.8	
Married, %	2 wk	118	91.5		140	90.7	
	6 wk	59	88.1		45	88.9	
Vaginal delivery, %	2 wk	118	95.8		140	94.3	
	6 wk	59	96.6		45	100	
BMI, kg/m^2^	2 wk	118	22.5	(20.8–24.1)	140	22.3	(20.8–24.3)
	6 wk	59	22.0	(20.6–24.1)	45	22.4	(21.1–24.1)
Weight, kg	2 wk	118	54.9	(50.4–60.0)	140	54.0	(49.8–58.4)
	6 wk	59	55.6	(49.1–61.2)	45	54.8	(50.8–59.5)
Height, cm	2 wk	118	157	(154–160)	140	155	(152–159)
	6 wk	59	157	(154–160)	45	156	(153–158)
Hemoglobin, g/L	2 wk	118	119	(110–131)	140	121	(110–131)
	6 wk	59	123	(117–132)	45	124	(116–132)
CD4 count, cells/μL	2 wk	108	452	(304–636)	129	485	(337–715)
	6 wk	51	524	(354–686)	41	503	(350–793)

^1^LNS, lipid-based supplement; IQR’ interquartile range.

### Changes in control group during lactation

#### Thiamin

**Fig 1:**
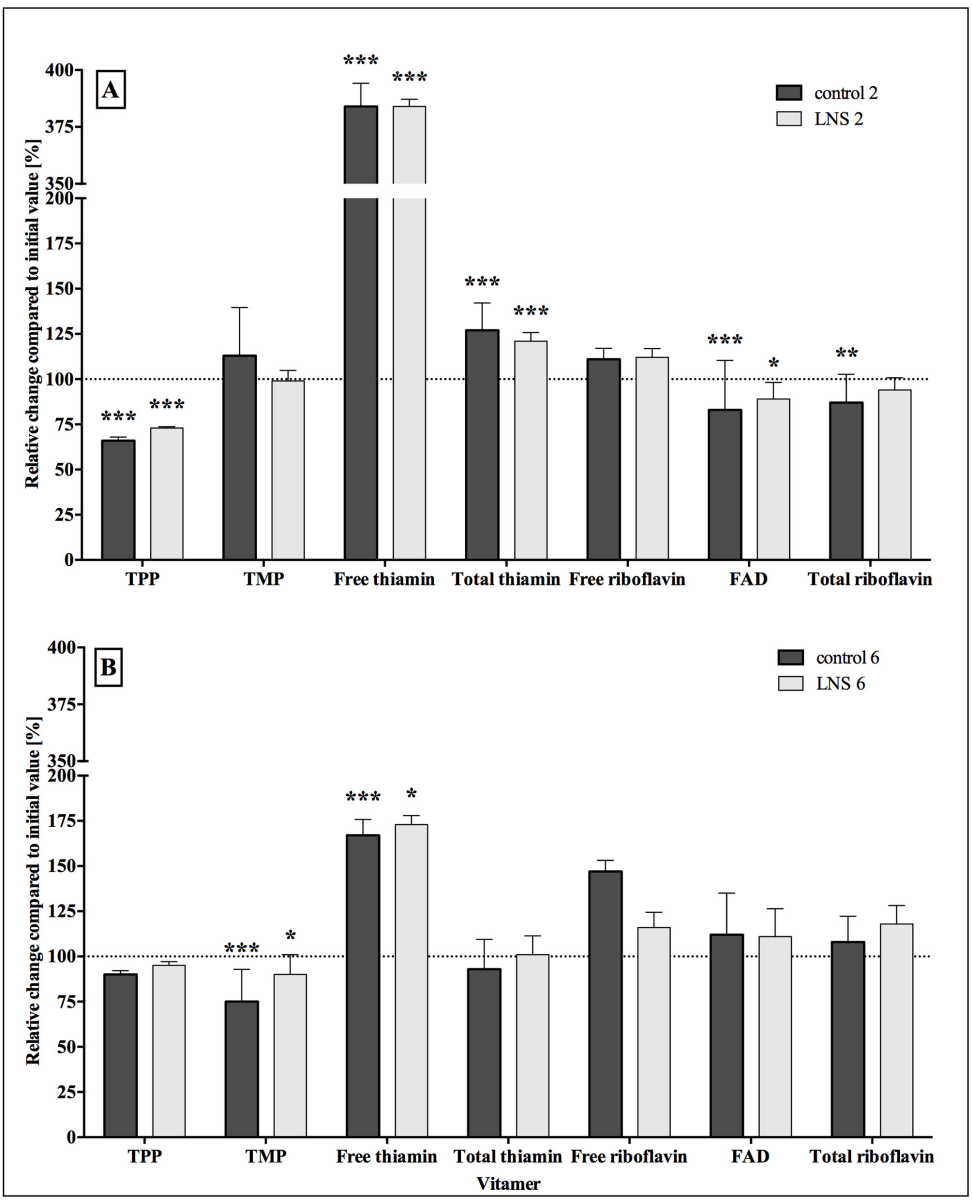
Percent change of concentrations and 95% confidence interval (CI) for each thiamin and riboflavin vitamier at 24 wk compared to the initial value (2 or 6 weeks, A and B respectively) by treatment group (control or LNS). Means were compared by paired t-test to evaluate changes over time within treatment and sub-set). Asterisks indicate level of significance compared to initial value (*, p < 0.01, **; p < 0.01; ***, p < 0.001). Control 2: control group initial value at 2 wk (n = 118). LNS 2: LNS group initial value at 2 wk (n = 140). Control 6: control group initial value at 6 wk (n = 59). LNS 6: LNS group initial value at 6 wk (n = 45). LNS, lipid-based nutrient supplement. TMP, thiamin-monophosphate. TPP, thiamin-pyrophosphate. FAD, flavin adenine dinucleotide.

Throughout lactation in the control group, TMP was the main form of thiamin, constituting 88% of total thiamin at 2 wk and 86% at 6 wk, falling to 71–72% at 24 weeks with a concomitant increase in free thiamin from 7–11% to 26–27% of the total (**Table 3**). In addition to the expected TMP and free thiamin, TPP, not detected in previous reports [14, 18, 19] was present in minor amounts, falling from 4.5% at 2 weeks to 2.5% at 24 wk of total thiamin (Table 3). Between 2 wk and 24 wk there was an increase in concentration of both free and total thiamin (both p < 0.001), while TPP concentrations fell (p < 0.001; **Fig 1A**). In contrast there was no change in TMP concentrations between 2 and 24 wk. Free thiamin amounts also increased between 6 and 24 wk, while total thiamin and TPP did not change and concentrations of TMP fell significantly (p <0.01; Fig 1B).

**Table 3:** Median relative contribution [%] to total thiamin and total riboflavin from each vitamer analyzed in milk from HIV-infected Malawian women, and change over time.

Treatment	Thiamin						Riboflavin			
	Free thiamin, *%*		TMP, *%*		TPP, *%*		Free riboflavin, *%*		FAD, *%*	
	2/6 wk^1^	24 wk	2/6 wk	24 wk	2/6 wk	24 wk	2/6 wk	24 wk	2/6 wk	24 wk
**Control**										
2 weeks	7.0	26.1	87.7	71.7	4.5	2.5	5.7	7.8	94.3	92.2
6 weeks	11.5	27.0	85.7	71.3	2.4	2.4	5.8	7.0	94.2	93.0
**LNS**										
2 weeks	8.4	29.9	87.5	66.7	3.7	2.5	11.6	16.7	88.4	83.3
6 weeks	11.5	21.3	85.9	76.8	1.9	2.3	14.8	14.7	85.2	85.3

^1^ Initial sample at 2 weeks or 6 weeks.

TMP, thiamin-monophosphate; TPP, thiamin-pyrophosphate; FAD, flavin adenine dinucleotide; LNS, lipid-based nutrient supplement. Supplementation started within 36 hours of delivery.

#### Riboflavin

The major form of riboflavin was FAD, which contributed about 94% of the total in the control group at 2 and 6 wk and a similar proportion at 24 wk, with free riboflavin making up the remainder (Table 3). From 2 to 24 wk the total riboflavin in milk fell by about 15% (p = 0.001) due to a reduction in FAD (p < 0.01) rather than free riboflavin **(**Fig 1). No significant changes in concentration were observed for free and total riboflavin or FAD from 6 to 24 wk in control samples (Fig 1).

### Effects of LNS

#### Thiamin

By 2 wk postpartum there were already positive effects of LNS supplementation on TMP, free and total thiamin compared to the control group (p < 0.05); there were no effects on TPP (**Table 4**). At 6 wk significant effects of LNS were not detected on TMP, free or total thiamin, although TPP was significantly lower in the LNS than the control group (p < 0.05). At 24 wk, there were no significant differences between concentrations of any of the vitamers or total thiamin concentrations as a result of thiamin supplementation. Free and total thiamin concentrations were substantially higher at 24 wk than earlier in lactation (Fig 1).

**Table 4:** Median concentration and interquartile range (IQR) of free thiamin, TMP, TPP, and total thiamin in breast milk of BAN study women assigned to one of two treatment arms within the two subgroups (initial sample at 2 or 6 weeks).

Vitamin^1^	Treatment Group				P value
	Control		LNS		
	n	median (IQR), *μg/L*	n	median (IQR), *μg/L*	
**Free Thiamin**					
2 wk	118	10.5 (6.5–17.5)	140	13.3 (8.2–20.7)	< 0.025
24 wk	118	40.4 (25.3–71)	140	51.2 (28.6–73)	n.s.
6 wk	59	24.5 (14.6–35.9)	45	23.4 (13.6–35.4)	n.s.
24 wk	59	40.9 (25.8–63)	45	40.4 (24.9–61)	n.s.
**TMP**					
2 wk	118	151 (119–188)	140	170 (141–202)	< 0.025
24 wk	118	177 (106–211)	140	169 (118–211)	n.s.
6 wk	59	204 (172–256)	45	208 (180–255)	n.s.
24 wk	59	153 (119–206)	45	187 (126–223)	n.s.
**TPP**					
2 wk	118	9.5 (6.6–15.3)	140	9.1 (6.6–13.1)	n.s.
24 wk	118	6.2 (3.6–9.9)	140	6.7 (4.2–9.8)	n.s.
6 wk	59	7.6 (5.0–12.6)	45	6.7 (4.9–8.4)	0.404
24 wk	59	6.8 (4.0–11.5)	45	6.3 (3.9–10.5)	n.s.
**Total Thiamin**					
2 wk	118	154 (118–191)	140	171 (137–201)	< 0.025
24 wk	118	196 (162–238)	140	207 (173–245)	n.s.
6 wk	59	220 (185–257)	45	213 (192–259)	n.s.
24 wk	59	205 (159–236)	45	214 (175–235)	n.s.

^1^Means were compared by t-test.

TMP, thiamin-monophosphate; TPP, thiamin-pyrophosphate; LNS, lipid-based nutrient supplement; n.s., not significant. Supplementation started within 36 hours of delivery.

#### Riboflavin

LNS significantly increased free and total riboflavin, but not FAD, in breast milk by 2 wk (both p < 0.001). Similar effects were also seen at 6 and 24 wk (**Table 5**): by 24 wk, total riboflavin in the LNS group was 42% (2 wk sub-set) and 32% (6wk sub-set) higher compared to the control group, as a result of increases in free riboflavin (p < 0.001) with increased FAD only for the 2 wk sub-set (p < 0.01, Table 4). In terms of changes across the 24 wk period in the LNS treatment group, from 2 to 24 wk there was a fall only in FAD (Table 4, p < 0.01) and none in free or total riboflavin. There were no significant changes in any vitamer from 6 to 24 wk.

**Table 5:** Median concentration and interquartile range of free riboflavin, FAD, and total riboflavin in breast milk of BAN study women assigned to one of two treatment arms within the two subgroups (initial sample at 2 or 6 weeks).

Vitamin^1^	Treatment Group				P value
	Control		LNS		
	n	median (IQR), *μg/L*	n	median (IQR), *μg/L*	
**Free Riboflavin**					
2 wk	118	6.3 (3.9–11.6)	140	17.5 (8.0–32.6)	< 0.0001
24 wk	118	7.0 (3.9–11.4)	140	19.6 (9.9–35.4)	< 0.0001
6 wk	59	5.0 (2.3–9.9)	45	14.8 (8.3–29.9)	<0.004
24 wk	59	7.4 (3.9–12.7)	45	17.2 (11.4–36.6)	<0.004
**FAD**					
2 wk	118	210 (152–282)	140	229 (164–295)	n.s.
24 wk	118	173 (128–221)	140	203 (147–275)	0.0021
6 wk	59	176 (119–272)	45	179 (148–247)	n.s.
24 wk	59	197 (131–244)	45	198 (141–283)	n.s.
**Total Riboflavin**					
2 wk	118	105 (78–148)	140	137 (97–188)	< 0.0001
24 wk	118	91 (69–122)	140	129 (88–166)	< 0.0001
6 wk	59	91 (62–146)	45	109 (84–158)	<0.004
24 wk	59	98 (68–131)	45	129 (90–181)	<0.004

^1^Means were compared by t-test.

FAD, flavin adenine dinucleotide; LNS, lipid-based nutrient supplement. Supplementation started within 36 hours of delivery.

## Discussion

### Thiamin

Free thiamin and TMP are the only vitamers reported to contribute to the total thiamin concentration in human milk [18, 19]. However, our method revealed that TPP constituted 1.9–4.5% of total thiamin in breast milk. While the method we developed follows the same sample preparation principle, HPLC-analyses done previously were carried out using isocratic mobile phase conditions, which could cause TPP to co-elute with nonspecific matrix components and therefore remain undetected. Using a mobile phase gradient and the flow rate as described above allowed separation of TPP from matrix interferences and subsequent identification by co-injecting a commercial standard. TPP usually represents only a minor contribution to total thiamin content, but we found that in some of the analyzed samples this vitamer contributed up to 40% of total thiamin.

Even though the milk used in this analysis was obtained from HIV-infected women, the breast milk concentrations of total thiamin showed comparable median concentrations to those of adequately nourished and apparently healthy mothers in other studies [14, 31, 32]. The value of 205 μg/L in the control group at 24 wk compares to the estimate of 210 μg/L from literature reports in the Dietary Reference Intakes [29]. Maternal thiamin supplementation resulted in increases in breast milk from about 54 μg/L to 150 μg/L (179.5–502.7 nmol/L) in severely depleted Cambodian women [14], but it is thought to be only transferred into milk to a limited degree [33]. Studies in the US showed no effect of thiamin supplementation on breast milk of adequately nourished mothers [31, 32]. Indeed, the only effect of LNS supplementation in this study of Malawian women was detected at 2 wk when there were significant increases in TMP and free thiamin concentrations compared to the control group. This effect was not observed at 24 wk or in the 6 wk sub-set indicating thiamin supplementation may be more effective in the early stages of lactation. Importantly, our data shows that milk collected at 2 wk will have substantially lower concentrations of total thiamin compared to milk collected at 6 wk (Table 4).

Details of thiamin uptake in the mammary gland are sparse. While free thiamin constitutes the transported form, the phosphorylated derivatives are the active co-enzymatic structure of the vitamin [34]. In the human body, phosphorylated thiamin derived from food undergoes de-phosphorylation prior to absorption in the intestinal lumen involving a specialized, pH-dependent, Na^+^-independent, carrier mediated system. Thiamin transporters *THTR1* and *THTR2* are both involved in the normal thiamin uptake process in human intestinal epithelial cells [35]. Both enzymes are members of the solute carrier family 19A, which also includes the reduced folate carrier *RFC-1* [36, 37]. Inside the cell, thiamin is converted into TPP by thiamin pyrophosphokinase-1 (TPK-1). TPP transport across the mitochondrial membrane is facilitated by thiamin pyrophosphate carrier (TPC), which is encoded by the SLC25A19 gene [38]. The lack of TPP in the cytosol indicates possible hydrolysis to TMP and subsequent recycling to free thiamin. While there are no enzymes reported for latter conversion, there is also no known intracellular role for TMP [39].

Whether these types of reactions are also located in the mammary gland is unknown. Water-soluble vitamin transport from the interstitial fluid into breast milk occurs mainly via the transcellular pathway, mediated by specific transporters that are located at the basolateral membrane, Golgi apparatus, secretory vesicles and apical membrane [40]. *THTR1* and *THTR2* have been identified and are active in breast tissue [41] but the expression of transporters from the SLC19 family in the actual mammary gland has yet not been described. No information is available about the transport mechanisms that mediate thiamin secretion into breast milk. The fact that milk concentrations are higher than plasma levels indicates active thiamin secretion into the milk [40]. The results obtained here reveal that free thiamin is the only vitamer showing a consistent significant increase in concentration in breast milk for both sub-sets over time and independently of supplementation, supporting the presence of an active secretion system, possibly of free thiamin. Nevertheless, TMP represents the main thiamin source in the milk; thus, free thiamin may be undergoing the above-described phosphorylation to TPP in the mammary gland, followed by secretion into breast milk and hydrolysis to TMP. Alternatively, phosphorylation of the free form to TMP might be possible; a direct secretion of TMP is also feasible [39]. Given that the amounts of phosphorylated vitamers either decreased or did not change over time, possible phosphorylation of thiamin, hydrolysis of TPP, or the secretion of TMP may be upregulated during the early stages of lactation. The only significant increase in TMP due to LNS occurred at 2 wk and initial TMP levels at 6 wk were significantly higher than the initial levels at 2 wk, supporting that possible targeted TMP production or transport may be peaking within the first weeks of lactation. However, even though there were no differences in the characteristics of study participants among the groups at their initial time point, the two sub-groups are independent from each other and differences in concentration could be due to the variation within each sub-set.

### Riboflavin

FAD and free riboflavin have been identified as the main contributors to total riboflavin in human milk with negligible amounts of other flavin derivatives [20, 21]. Riboflavin supplementation has been shown to be reflected in increased milk concentration [33, 42], which was also the case in the present study. Supplementation with about 1 x RDA resulted in a 2–3 fold increase of free riboflavin, but FAD levels were increased only at 24 wk in the 2 wk sub-set when compared to the control group. However, total riboflavin concentrations in the LNS compared to the control group were greatly increased at all time points. Given that free riboflavin is generally used in supplements the greater increase in this vitamer can be expected, suggesting its efficient absorption and transport into milk rather than conversion into its co-enzymatic form prior to secretion. However, less than 10% of all samples analyzed revealed adequate levels of total riboflavin compared to the AI value of 350 μg/L [29] or reached levels comparable to those from well-nourished mothers [21, 43]. The median values of 91 and 98 μg/L in the control group at 24 wk (both sub-sets) compare unfavorably to the 350 μg/L of total riboflavin assumed for establishing the AI for infants [29]. Even after 24 wk of supplementation the median total riboflavin concentration was only 129 μg/L, and more than 98% of samples were below AI values. The FAD:riboflavin-proportion of 92–94%:6–8% observed in the controls was also seen in well-nourished Japanese mothers [21], indicating similar secretion patterns independently of maternal riboflavin and health status.

Transport of riboflavin into breast milk has been linked to breast cancer resistance protein (*BCRP*), a highly conserved member of the ATP-binding cassette (ABC) transporter superfamily [11, 44]. This multidrug transporter has a broad substrate specificity and in addition to riboflavin, it actively extrudes drugs, carcinogens, and dietary toxins from cells [45]. During pregnancy and lactation, *BCRP* is strongly induced and actively transports free riboflavin but also toxins into breast milk via an ATP-dependent mechanism. FAD was present in milk at similar levels in *BCRP1*^*-/-*^ knockout compared to wild-type mice indicating the presence of a *BCRP*-independent, possibly active transport of this vitamer into the milk [11].

We found consistent concentrations of free riboflavin in breast milk throughout lactation for both the 2 and 6 wk sub-set independently of treatment, suggesting a steady secretion and supply of the vitamer. In contrast, FAD amounts decreased significantly from 2 to 24 weeks, but there was no significant change observed in the 6 wk sub-set. This was true for both the control and the supplemented group. That the contributions of FAD remained between 92 to 94% and 83 to 88% in the control and LNS group respectively also suggests a steady FAD supply in the milk over time with a shift in contribution when supplements were taken, making free riboflavin the driving force for the change in total riboflavin content, which may induce upregulation of *BCRP* expression.

## Conclusion

Of all the thiamin vitamers present in human milk, only free thiamin concentrations increased during lactation suggesting an active transport of this vitamer into the mammary gland. The main vitamer TMP may be also actively transported into the milk, but could also derive from phosphorylation of free thiamin or hydrolysis of TPP. These transport, phosphorylation, or hydrolysis mechanisms may be upregulated during the early stages of lactation. LNS supplementation only positively affected thiamin and TMP concentrations, and only in the first 2 wk postpartum. TPP has been identified as a contributor to total thiamin in breast milk for the first time, with contributions in some cases up to 40%. Free riboflavin concentrations remain comparable throughout lactation, while FAD amounts decline from 2 to 24 wk. Supplementation is reflected in an increased milk concentration of free riboflavin, but not necessarily in FAD suggesting a preferred secretion of the free form into breast milk by *BCRP*. Active FAD secretion most likely occurs by a BCRP-independent and FAD specific but unknown mechanism. Further studies are needed to gain better insight into the mechanisms involved in vitamin secretion into human milk, the triggers initiating vitamer conversion in the mammary gland, and the roles of the different forms of the vitamins.

## Supporting Information

S1 Table Concentrations of TPP, TMP, thiamin, total thiamin, riboflavin, FAD, and total riboflavin [μg/L] in the control group at 2/6 weeks. (DOCX)

S2 Table Concentrations of TPP, TMP, thiamin, total thiamin, riboflavin, FAD, and total riboflavin [μg/L] in the LNS group at 2/6 weeks. (DOCX)

S3 Table Concentrations of TPP, TMP, thiamin, total thiamin, riboflavin, FAD, and total riboflavin [μg/L] in the control group at 24 weeks. (DOCX)

S4 Table Concentrations of TPP, TMP, thiamin, total thiamin, riboflavin, FAD, and total riboflavin [μg/L] in the LNS group at 24 weeks. (DOCX)
