# Supplementary material for: Thiamin and Riboflavin in Human Milk: Effects of Lipid-Based Nutrient Supplementation and Stage of Lactation on Vitamer Secretion and Contributions to Total Vitamin Content
Source: PLoS One. 2016 Feb 17;11(2):e0149479. doi: 10.1371/journal.pone.0149479 (PMC4757446; doi:10.1371/journal.pone.0149479)
Supplement: S1 Table — (DOCX) [file pone.0149479.s001.docx]

**S1 Table:** Concentrations of TPP, TMP, thiamin, total thiamin, riboflavin, FAD, and total riboflavin [µg/L] in the control group at 2/6 weeks.

| **Sample** | **time of collection [wk]** | **TPP** | **TMP** | **thiamin** | **total thiamin** | **riboflavin** | **FAD** | **total riboflavin** |
| --- | --- | --- | --- | --- | --- | --- | --- | --- |
| 1 | 6 | 6.74 | 149.58 | 71.51 | 206.57 | 1.81 | 84.92 | 42.49 |
| 2 | 6 | 4.57 | 260.39 | 31.14 | 261.19 | 4.43 | 137.52 | 70.31 |
| 3 | 2 | 9.00 | 137.65 | 5.45 | 131.72 | 13.63 | 359.24 | 185.74 |
| 4 | 2 | 10.11 | 35.12 | 0.89 | 38.62 | 1.95 | 212.58 | 103.80 |
| 5 | 6 | 11.42 | 203.48 | 34.95 | 220.26 | 3.57 | 123.85 | 62.91 |
| 6 | 2 | 4.95 | 188.00 | 13.93 | 181.19 | 26.69 | 293.98 | 167.53 |
| 7 | 2 | 14.82 | 154.46 | 10.46 | 155.49 | 2.71 | 332.31 | 161.92 |
| 8 | 6 | 16.97 | 172.28 | 67.86 | 229.93 | 7.55 | 324.88 | 163.20 |
| 9 | 2 | 9.93 | 127.73 | 7.00 | 125.28 | 7.19 | 301.21 | 151.50 |
| 10 | 2 | 15.65 | 109.86 | 3.23 | 110.00 | 6.34 | 323.51 | 161.33 |
| 11 | 6 | 23.82 | 231.00 | 32.01 | 250.07 | 8.83 | 283.88 | 144.83 |
| 12 | 2 | 30.93 | 263.75 | 15.88 | 267.49 | 1.63 | 375.06 | 181.33 |
| 13 | 2 | 19.31 | 150.51 | 23.01 | 167.77 | 22.81 | 508.31 | 266.34 |
| 14 | 2 | 7.22 | 122.79 | 8.60 | 120.67 | 15.14 | 255.21 | 137.41 |
| 15 | 6 | 18.37 | 189.98 | 53.78 | 232.26 | 11.78 | 325.14 | 167.56 |
| 16 | 2 | 9.19 | 111.25 | 7.18 | 110.58 | 8.19 | 216.46 | 111.89 |
| 17 | 2 | 11.72 | 112.04 | 5.84 | 111.72 | 11.58 | 193.25 | 104.17 |
| 18 | 2 | 7.79 | 190.56 | 8.74 | 180.24 | 12.27 | 338.97 | 174.67 |
| 19 | 2 | 4.35 | 120.87 | 8.12 | 116.49 | 5.75 | 236.50 | 119.05 |
| 20 | 2 | 7.78 | 137.02 | 7.12 | 131.98 | 8.47 | 220.61 | 114.16 |
| 21 | 6 | 10.65 | 206.52 | 15.83 | 203.26 | 10.71 | 274.79 | 142.36 |
| 22 | 2 | 4.77 | 190.44 | 8.23 | 177.48 | 2.63 | 208.61 | 102.58 |
| 23 | 6 | 3.60 | 236.76 | 8.97 | 217.75 | 2.17 | 81.11 | 41.03 |
| 24 | 6 | 12.34 | 191.44 | 36.80 | 212.29 | 16.19 | 250.96 | 136.43 |
| 25 | 6 | 6.10 | 257.25 | 22.36 | 250.75 | 5.83 | 224.60 | 113.44 |
| 26 | 6 | 3.55 | 107.61 | 8.47 | 104.71 | 3.75 | 117.30 | 59.95 |
| 27 | 2 | 9.48 | 201.92 | 10.32 | 192.91 | 2.91 | 199.39 | 98.44 |
| 28 | 2 | 12.67 | 230.49 | 26.42 | 236.15 | 9.54 | 132.68 | 73.11 |
| 29 | 2 | 12.91 | 131.62 | 8.83 | 132.61 | 10.29 | 227.35 | 119.21 |
| 30 | 2 | 8.01 | 236.62 | 29.03 | 240.81 | 11.02 | 417.67 | 211.13 |
| 31 | 2 | 16.66 | 137.12 | 7.41 | 138.64 | 11.95 | 303.67 | 157.44 |
| 32 | 2 | 10.65 | 155.97 | 9.38 | 152.77 | 2.17 | 137.36 | 67.98 |
| 33 | 6 | 7.24 | 204.67 | 11.67 | 195.07 | 6.59 | 175.50 | 90.67 |
| 34 | 6 | 5.42 | 222.11 | 23.72 | 221.02 | 2.76 | 149.88 | 74.56 |
| 35 | 6 | 7.10 | 336.81 | 23.43 | 321.83 | 2.03 | 196.67 | 96.25 |
| 36 | 2 | 24.13 | 152.42 | 10.81 | 160.65 | 2.22 | 110.81 | 55.31 |
| 37 | 2 | 10.61 | 108.08 | 6.83 | 108.47 | 10.06 | 431.12 | 216.60 |
| 38 | 2 | 4.74 | 166.34 | 12.09 | 160.33 | 7.20 | 228.01 | 116.44 |
| 39 | 2 | 18.01 | 82.88 | 4.96 | 89.89 | 6.76 | 260.05 | 131.35 |
| 40 | 2 | 8.92 | 228.91 | 9.55 | 215.26 | 3.08 | 218.33 | 107.68 |
| 41 | 2 | 5.16 | 98.31 | 8.09 | 97.37 | 1.54 | 239.37 | 116.22 |
| 42 | 6 | 10.00 | 187.57 | 34.28 | 204.73 | 5.38 | 147.99 | 76.28 |
| 43 | 2 | 9.49 | 218.75 | 9.13 | 206.39 | 4.49 | 210.36 | 105.27 |
| 44 | 2 | 6.91 | 101.46 | 6.60 | 99.86 | 5.67 | 407.67 | 200.98 |
| 45 | 2 | 11.23 | 156.62 | 7.48 | 151.85 | 5.82 | 321.29 | 159.76 |
| 46 | 2 | 7.95 | 117.86 | 12.60 | 120.89 | 3.07 | 155.29 | 77.47 |
| 47 | 2 | 8.34 | 134.90 | 6.05 | 129.46 | 6.07 | 306.00 | 152.67 |
| 48 | 6 | 6.28 | 179.51 | 41.33 | 202.14 | 6.87 | 194.54 | 100.07 |
| 49 | 2 | 5.34 | 100.72 | 2.24 | 93.75 | 3.89 | 161.46 | 81.25 |
| 50 | 2 | 20.71 | 129.10 | 17.17 | 144.27 | 5.20 | 282.63 | 140.61 |
| 51 | 6 | 22.35 | 240.91 | 31.62 | 257.27 | 12.04 | 410.87 | 208.89 |
| 52 | 2 | 9.77 | 127.91 | 11.94 | 130.27 | 26.65 | 291.43 | 166.28 |
| 53 | 2 | 17.24 | 169.66 | 25.42 | 185.39 | 4.90 | 330.67 | 163.32 |
| 54 | 6 | 5.15 | 294.46 | 21.34 | 281.48 | 2.22 | 216.22 | 105.82 |
| 55 | 2 | 10.13 | 128.81 | 3.72 | 123.08 | 4.64 | 175.42 | 88.68 |
| 56 | 2 | 4.73 | 116.63 | 10.61 | 115.55 | 9.14 | 232.54 | 120.55 |
| 57 | 6 | 11.59 | 122.35 | 29.42 | 144.19 | 11.53 | 350.39 | 179.40 |
| 58 | 2 | 40.08 | 172.23 | 32.79 | 211.16 | 23.79 | 294.79 | 165.02 |
| 59 | 2 | 7.48 | 139.35 | 4.74 | 131.41 | 5.39 | 402.77 | 198.36 |
| 60 | 2 | 9.25 | 174.80 | 17.53 | 176.33 | 16.45 | 226.70 | 125.06 |
| 61 | 6 | 22.34 | 46.79 | 12.75 | 69.31 | 16.41 | 348.56 | 183.41 |
| 62 | 2 | 6.53 | 162.45 | 10.22 | 156.34 | 16.02 | 265.12 | 143.04 |
| 63 | 2 | 5.84 | 232.44 | 14.91 | 221.51 | 24.81 | 203.33 | 122.22 |
| 64 | 2 | 41.24 | 78.59 | 14.12 | 111.75 | 6.42 | 404.76 | 200.34 |
| 65 | 6 | 4.81 | 261.02 | 8.75 | 239.51 | 3.62 | 200.99 | 99.91 |
| 66 | 6 | 8.90 | 254.14 | 33.31 | 260.98 | 1.76 | 153.60 | 75.35 |
| 67 | 2 | 6.50 | 93.88 | 4.51 | 90.88 | 6.21 | 264.06 | 132.72 |
| 68 | 2 | 9.16 | 110.91 | 3.54 | 106.62 | 9.26 | 225.04 | 117.08 |
| 69 | 2 | 8.53 | 203.02 | 7.86 | 190.73 | 10.58 | 351.04 | 178.76 |
| 70 | 6 | 3.29 | 318.89 | 12.09 | 292.19 | 5.02 | 132.15 | 68.33 |
| 71 | 6 | 6.90 | 140.90 | 93.21 | 220.82 | 4.08 | 170.66 | 85.84 |
| 72 | 6 | 20.15 | 259.31 | 24.50 | 264.63 | 13.95 | 245.35 | 131.50 |
| 73 | 2 | 25.70 | 169.08 | 24.41 | 189.87 | 7.31 | 302.34 | 152.17 |
| 74 | 6 | 6.65 | 56.30 | 121.55 | 175.30 | 1.90 | 205.17 | 100.19 |
| 75 | 6 | 12.43 | 300.07 | 37.64 | 307.80 | 3.36 | 142.50 | 71.63 |
| 76 | 2 | 11.76 | 84.10 | 9.84 | 91.41 | 4.31 | 163.35 | 82.57 |
| 77 | 2 | 22.68 | 218.56 | 44.05 | 250.47 | 67.62 | 308.89 | 215.61 |
| 78 | 2 | 6.36 | 160.79 | 11.34 | 155.89 | 12.45 | 245.59 | 130.12 |
| 79 | 2 | 4.89 | 122.83 | 8.88 | 119.33 | 13.78 | 107.01 | 65.05 |
| 80 | 2 | 6.64 | 199.49 | 8.18 | 186.65 | 14.85 | 234.64 | 127.27 |
| 81 | 2 | 8.73 | 128.23 | 2.75 | 120.61 | 5.72 | 229.23 | 115.55 |
| 82 | 2 | 5.66 | 221.97 | 14.69 | 212.04 | 4.46 | 236.40 | 117.72 |
| 83 | 6 | 4.68 | 192.43 | 15.14 | 186.07 | 10.83 | 370.61 | 188.39 |
| 84 | 2 | 10.00 | 204.50 | 16.50 | 201.70 | 26.61 | 418.64 | 227.18 |
| 85 | 2 | 20.42 | 260.63 | 21.38 | 262.85 | 10.86 | 234.67 | 123.29 |
| 86 | 2 | 3.47 | 86.46 | 5.41 | 83.17 | 12.86 | 338.85 | 175.20 |
| 87 | 2 | 18.66 | 127.67 | 14.14 | 138.55 | 5.39 | 162.50 | 83.25 |
| 88 | 2 | 14.96 | 194.55 | 19.96 | 200.01 | 17.41 | 172.97 | 100.28 |
| 89 | 6 | 6.44 | 339.88 | 21.75 | 322.37 | 7.95 | 119.66 | 65.28 |
| 90 | 2 | 26.15 | 152.50 | 29.58 | 180.91 | 6.89 | 155.66 | 81.47 |
| 91 | 2 | 6.64 | 225.39 | 24.10 | 225.12 | 28.96 | 245.47 | 146.57 |
| 92 | 2 | 11.19 | 45.16 | 0.79 | 48.04 | 6.57 | 267.67 | 134.81 |
| 93 | 2 | 10.61 | 132.59 | 10.86 | 133.85 | 24.56 | 240.95 | 140.00 |
| 94 | 2 | 4.30 | 175.51 | 15.73 | 171.65 | 3.58 | 125.85 | 63.87 |
| 95 | 2 | 7.49 | 176.73 | 11.10 | 170.34 | 7.69 | 181.94 | 94.85 |
| 96 | 2 | 5.92 | 152.36 | 9.50 | 146.41 | 9.13 | 158.12 | 84.88 |
| 97 | 6 | 4.94 | 162.46 | 25.22 | 170.23 | 3.78 | 109.36 | 56.17 |
| 98 | 6 | 5.12 | 189.89 | 16.29 | 185.31 | 1.69 | 137.73 | 67.68 |
| 99 | 2 | 7.06 | 207.65 | 15.36 | 201.23 | 14.36 | 118.59 | 71.18 |
| 100 | 6 | 13.18 | 161.52 | 36.32 | 186.33 | 7.31 | 445.91 | 220.94 |
| 101 | 2 | 19.44 | 146.39 | 21.09 | 162.35 | 2.12 | 90.42 | 45.44 |
| 102 | 6 | 3.03 | 252.81 | 36.99 | 259.34 | 9.08 | 63.39 | 39.45 |
| 103 | 2 | 9.42 | 170.42 | 15.31 | 170.41 | 14.52 | 146.72 | 84.81 |
| 104 | 2 | 18.42 | 162.07 | 21.74 | 175.93 | 13.53 | 175.57 | 97.65 |
| 105 | 6 | 7.99 | 94.69 | 6.49 | 94.62 | 0.28 | 106.05 | 51.09 |
| 106 | 2 | 6.16 | 273.70 | 14.65 | 257.42 | 23.35 | 232.06 | 134.53 |
| 107 | 6 | 12.98 | 296.86 | 42.44 | 310.21 | 7.88 | 172.35 | 90.45 |
| 108 | 2 | 9.98 | 180.65 | 17.11 | 181.52 | 1.10 | 135.80 | 66.16 |
| 109 | 2 | 5.23 | 166.41 | 6.08 | 154.73 | 3.89 | 38.57 | 22.37 |
| 110 | 6 | 5.04 | 179.79 | 7.90 | 168.08 | 0.00 | 33.41 | 16.01 |
| 111 | 2 | 10.18 | 160.34 | 12.89 | 159.75 | 4.70 | 212.86 | 106.68 |
| 112 | 2 | 27.96 | 223.90 | 25.87 | 240.67 | 0.85 | 107.86 | 52.52 |
| 113 | 2 | 8.51 | 167.55 | 19.37 | 171.33 | 7.36 | 228.25 | 116.71 |
| 114 | 2 | 3.96 | 141.07 | 17.74 | 143.42 | 6.83 | 113.56 | 61.24 |
| 115 | 2 | 7.26 | 125.57 | 6.51 | 121.02 | 0.54 | 75.57 | 36.75 |
| 116 | 2 | 10.83 | 188.34 | 13.08 | 184.79 | 11.63 | 154.88 | 85.83 |
| 117 | 2 | 10.54 | 120.40 | 4.31 | 116.63 | 2.30 | 135.90 | 67.41 |
| 118 | 2 | 5.77 | 141.72 | 7.04 | 134.56 | 0.19 | 151.72 | 72.88 |
| 119 | 6 | 13.41 | 235.55 | 8.40 | 223.06 | 5.46 | 90.96 | 49.04 |
| 120 | 2 | 33.38 | 122.26 | 21.91 | 152.02 | 1.74 | 99.75 | 49.53 |
| 121 | 2 | 7.86 | 141.59 | 7.02 | 135.91 | 0.95 | 156.52 | 75.93 |
| 122 | 6 | 4.39 | 199.70 | 33.43 | 210.49 | 1.14 | 163.83 | 79.63 |
| 123 | 6 | 6.71 | 175.90 | 18.32 | 176.28 | 1.44 | 134.72 | 65.98 |
| 124 | 2 | 16.01 | 84.23 | 4.83 | 89.52 | 15.96 | 368.11 | 192.32 |
| 125 | 2 | 15.36 | 157.63 | 42.81 | 190.98 | 11.33 | 275.36 | 143.25 |
| 126 | 2 | 6.12 | 179.19 | 23.35 | 183.76 | 23.12 | 144.67 | 92.44 |
| 127 | 2 | 5.77 | 146.68 | 10.68 | 142.54 | 2.42 | 127.95 | 63.72 |
| 128 | 2 | 7.17 | 189.87 | 26.06 | 196.53 | 2.86 | 199.00 | 98.21 |
| 129 | 2 | 16.26 | 64.24 | 5.13 | 72.59 | 4.54 | 183.39 | 92.41 |
| 130 | 6 | 11.57 | 87.91 | 49.17 | 133.93 | 2.02 | 61.06 | 31.27 |
| 131 | 2 | 9.44 | 39.48 | 1.62 | 42.69 | 4.48 | 148.49 | 75.62 |
| 132 | 2 | 63.42 | 179.19 | 51.82 | 252.76 | 5.22 | 191.87 | 97.15 |
| 133 | 2 | 9.06 | 184.78 | 17.24 | 184.60 | 8.38 | 191.30 | 100.03 |
| 134 | 2 | 4.61 | 247.54 | 9.51 | 228.39 | 4.08 | 60.48 | 33.05 |
| 135 | 6 | 4.67 | 151.34 | 16.15 | 151.27 | 4.48 | 82.38 | 43.95 |
| 136 | 2 | 6.67 | 188.46 | 40.65 | 209.53 | 2.29 | 81.42 | 41.30 |
| 137 | 2 | 18.20 | 137.53 | 23.64 | 156.30 | 5.66 | 197.67 | 100.36 |
| 138 | 2 | 14.91 | 163.19 | 7.04 | 159.74 | 1.82 | 159.05 | 78.02 |
| 139 | 6 | 7.56 | 176.57 | 26.39 | 185.53 | 1.27 | 55.74 | 27.98 |
| 140 | 2 | 9.99 | 193.92 | 17.37 | 193.35 | 5.78 | 109.77 | 58.37 |
| 141 | 2 | 23.43 | 168.22 | 18.33 | 181.43 | 4.23 | 182.27 | 91.56 |
| 142 | 2 | 15.06 | 260.69 | 25.19 | 262.92 | 7.15 | 333.61 | 166.98 |
| 143 | 2 | 57.32 | 166.36 | 62.88 | 248.34 | 15.65 | 277.96 | 148.82 |
| 144 | 6 | 4.28 | 171.05 | 14.27 | 166.29 | 2.96 | 299.24 | 146.33 |
| 145 | 2 | 6.64 | 125.24 | 3.63 | 117.41 | 12.67 | 340.84 | 175.97 |
| 146 | 2 | 2.54 | 95.38 | 4.12 | 88.99 | 4.15 | 48.71 | 27.48 |
| 147 | 6 | 8.82 | 270.50 | 35.52 | 277.38 | 3.34 | 217.57 | 107.58 |
| 148 | 2 | 3.29 | 231.31 | 15.52 | 219.34 | 4.83 | 176.96 | 89.61 |
| 149 | 2 | 5.81 | 118.17 | 3.88 | 110.93 | 10.88 | 126.70 | 71.58 |
| 150 | 2 | 14.21 | 48.55 | 1.90 | 54.25 | 14.17 | 583.72 | 293.83 |
| 151 | 2 | 11.34 | 120.88 | 8.61 | 121.92 | 3.85 | 130.98 | 66.61 |
| 152 | 6 | 12.85 | 109.96 | 5.97 | 110.84 | 1.88 | 151.67 | 74.55 |
| 153 | 2 | 8.35 | 42.75 | 2.36 | 45.50 | 1.98 | 93.40 | 46.72 |
| 154 | 6 | 4.53 | 239.94 | 16.80 | 229.00 | 2.45 | 59.16 | 30.80 |
| 155 | 6 | 10.49 | 188.56 | 13.75 | 185.41 | 2.41 | 196.76 | 96.68 |
| 156 | 2 | 17.19 | 50.49 | 3.87 | 60.01 | 3.09 | 124.83 | 62.90 |
| 157 | 2 | 18.79 | 194.82 | 11.57 | 194.56 | 5.49 | 128.92 | 67.26 |
| 158 | 6 | 33.15 | 210.27 | 35.42 | 242.02 | 11.02 | 316.34 | 162.58 |
| 159 | 2 | 6.45 | 135.51 | 6.24 | 128.84 | 2.35 | 203.59 | 99.89 |
| 160 | 6 | 14.36 | 290.77 | 27.85 | 291.29 | 5.57 | 344.74 | 170.74 |
| 161 | 2 | 5.85 | 63.28 | 2.12 | 61.38 | 2.64 | 186.61 | 92.04 |
| 162 | 6 | 7.34 | 266.29 | 14.94 | 252.09 | 166.53 | 242.49 | 282.71 |
| 163 | 2 | 7.41 | 100.29 | 3.99 | 96.58 | 6.98 | 153.40 | 80.48 |
| 164 | 6 | 9.22 | 203.75 | 36.61 | 220.60 | 83.34 | 195.32 | 176.91 |
| 165 | 2 | 3.15 | 41.52 | 2.48 | 40.87 | 2.12 | 54.26 | 28.11 |
| 166 | 2 | 11.09 | 222.07 | 33.09 | 234.36 | 2.23 | 81.13 | 41.10 |
| 167 | 2 | 14.60 | 116.46 | 25.86 | 137.63 | 5.45 | 159.70 | 81.97 |
| 168 | 2 | 26.82 | 106.13 | 13.81 | 125.23 | 4.04 | 182.75 | 91.60 |
| 169 | 6 | 24.26 | 322.65 | 45.61 | 343.81 | 37.89 | 433.86 | 245.75 |
| 170 | 6 | 4.10 | 190.79 | 16.49 | 185.58 | 2.51 | 104.96 | 52.80 |
| 171 | 6 | 8.18 | 231.72 | 100.10 | 307.73 | 16.26 | 255.74 | 138.79 |
| 172 | 6 | 4.77 | 139.34 | 5.40 | 130.15 | 5.62 | 116.46 | 61.41 |
| 173 | 2 | 18.78 | 194.10 | 31.08 | 213.43 | 10.61 | 314.20 | 161.14 |
| 174 | 6 | 15.82 | 127.39 | 15.86 | 138.02 | 19.47 | 409.67 | 215.74 |
| 175 | 6 | 3.47 | 210.86 | 6.52 | 192.65 | 0.09 | 88.46 | 42.47 |
| 176 | 6 | 9.40 | 272.80 | 12.40 | 256.67 | 6.93 | 270.05 | 136.31 |
| 177 | 6 | 23.50 | 212.99 | 26.14 | 228.30 | 39.35 | 382.66 | 222.69 |

TPP: thiamin pyrophosphate, TMP: thiamin monophosphate, FAD: flavin adenine dinucleotide
