# Supplementary material for: Thiamin and Riboflavin in Human Milk: Effects of Lipid-Based Nutrient Supplementation and Stage of Lactation on Vitamer Secretion and Contributions to Total Vitamin Content
Source: PLoS One. 2016 Feb 17;11(2):e0149479. doi: 10.1371/journal.pone.0149479 (PMC4757446; doi:10.1371/journal.pone.0149479)
Supplement: S2 Table — (DOCX) [file pone.0149479.s002.docx]

**S2 Table:** Concentrations of TPP, TMP, thiamin, total thiamin, riboflavin, FAD, and total riboflavin [µg/L] in the LNS group at 2/6 weeks.

| **Sample** | **time of collection [wk]** | **TPP** | **TMP** | **thiamin** | **total thiamin** | **riboflavin** | **FAD** | **total riboflavin** |
| --- | --- | --- | --- | --- | --- | --- | --- | --- |
| 1 | 2 | 12.73 | 186.16 | 29.70 | 200.86 | 21.96 | 380.95 | 204.48 |
| 2 | 2 | 7.42 | 182.31 | 8.21 | 172.26 | 5.79 | 214.16 | 108.40 |
| 3 | 2 | 13.91 | 196.15 | 15.36 | 196.06 | 16.86 | 393.41 | 205.35 |
| 4 | 2 | 29.08 | 118.24 | 11.92 | 135.48 | 8.61 | 379.20 | 190.28 |
| 5 | 2 | 2.85 | 269.78 | 13.04 | 250.05 | 9.62 | 422.44 | 212.01 |
| 6 | 2 | 10.26 | 67.06 | 94.54 | 160.22 | 183.20 | 386.00 | 368.13 |
| 7 | 2 | 7.31 | 103.48 | 4.80 | 100.11 | 11.11 | 285.49 | 147.88 |
| 8 | 2 | 11.66 | 187.61 | 23.26 | 194.93 | 21.43 | 252.62 | 142.46 |
| 9 | 2 | 9.88 | 207.17 | 14.17 | 201.62 | 82.60 | 263.27 | 208.73 |
| 10 | 2 | 25.49 | 23.18 | 8.00 | 46.23 | 23.42 | 428.21 | 228.58 |
| 11 | 2 | 10.59 | 152.82 | 13.35 | 153.95 | 7.78 | 166.19 | 87.40 |
| 12 | 2 | 14.81 | 179.35 | 10.44 | 177.14 | 32.66 | 337.91 | 194.56 |
| 13 | 2 | 13.37 | 166.05 | 17.28 | 171.37 | 5.78 | 267.45 | 133.91 |
| 14 | 2 | 19.93 | 124.76 | 11.70 | 134.47 | 15.02 | 239.34 | 129.68 |
| 15 | 2 | 9.25 | 178.63 | 5.64 | 167.78 | 6.55 | 303.35 | 151.89 |
| 16 | 2 | 4.24 | 181.72 | 7.10 | 168.39 | 3.20 | 209.77 | 103.70 |
| 17 | 2 | 3.40 | 297.91 | 36.71 | 298.61 | 331.06 | 199.50 | 426.64 |
| 18 | 2 | 8.22 | 252.03 | 13.00 | 238.34 | 36.88 | 200.31 | 132.84 |
| 19 | 2 | 8.53 | 84.15 | 5.40 | 84.74 | 5.89 | 293.90 | 146.70 |
| 20 | 2 | 9.46 | 188.65 | 8.06 | 179.08 | 7.20 | 115.78 | 62.68 |
| 21 | 2 | 7.26 | 152.82 | 4.64 | 142.89 | 21.32 | 358.45 | 193.05 |
| 22 | 2 | 3.68 | 202.61 | 3.06 | 182.15 | 0.00 | 54.12 | 25.93 |
| 23 | 2 | 6.55 | 170.23 | 12.32 | 165.23 | 21.57 | 291.19 | 161.08 |
| 24 | 2 | 3.78 | 191.63 | 5.70 | 175.30 | 17.57 | 297.03 | 159.87 |
| 25 | 2 | 8.61 | 189.38 | 12.63 | 183.67 | 21.26 | 228.86 | 130.91 |
| 26 | 2 | 40.71 | 93.08 | 17.42 | 127.29 | 5.21 | 124.80 | 65.01 |
| 27 | 2 | 5.13 | 170.43 | 8.37 | 160.46 | 15.59 | 252.83 | 136.72 |
| 28 | 2 | 27.92 | 166.15 | 30.40 | 194.87 | 40.39 | 275.89 | 172.57 |
| 29 | 2 | 11.31 | 153.52 | 9.02 | 150.74 | 83.10 | 335.29 | 243.74 |
| 30 | 2 | 10.23 | 186.39 | 6.38 | 175.97 | 17.75 | 395.74 | 207.35 |
| 31 | 2 | 10.70 | 222.54 | 9.32 | 210.73 | 9.20 | 252.54 | 130.19 |
| 32 | 2 | 11.70 | 157.52 | 14.14 | 159.62 | 17.08 | 308.81 | 165.03 |
| 33 | 2 | 8.25 | 144.45 | 11.98 | 143.64 | 30.46 | 433.45 | 238.13 |
| 34 | 2 | 22.09 | 223.85 | 20.65 | 231.26 | 8.06 | 443.50 | 220.54 |
| 35 | 2 | 8.36 | 210.38 | 21.62 | 210.78 | 23.67 | 175.84 | 107.92 |
| 36 | 2 | 23.80 | 168.40 | 19.58 | 183.11 | 33.22 | 333.85 | 193.17 |
| 37 | 2 | 9.79 | 125.07 | 14.70 | 130.57 | 7.16 | 351.91 | 175.75 |
| 38 | 2 | 12.39 | 155.47 | 8.23 | 152.42 | 23.10 | 189.09 | 113.70 |
| 39 | 2 | 6.47 | 284.64 | 23.71 | 276.22 | 39.29 | 323.46 | 194.25 |
| 40 | 2 | 8.24 | 177.15 | 11.49 | 171.62 | 8.37 | 149.73 | 80.11 |
| 41 | 2 | 16.66 | 205.69 | 29.93 | 220.88 | 45.19 | 440.18 | 256.09 |
| 42 | 2 | 9.17 | 187.33 | 29.61 | 199.27 | 25.53 | 276.18 | 157.85 |
| 43 | 2 | 6.59 | 115.29 | 4.24 | 109.32 | 10.78 | 270.22 | 140.24 |
| 44 | 2 | 7.44 | 172.57 | 15.45 | 171.03 | 10.55 | 277.34 | 143.42 |
| 45 | 2 | 2.45 | 119.02 | 27.21 | 132.61 | 13.54 | 175.67 | 97.71 |
| 46 | 2 | 13.17 | 124.10 | 16.33 | 133.74 | 20.94 | 383.05 | 204.46 |
| 47 | 2 | 7.31 | 170.07 | 12.06 | 165.37 | 6.73 | 337.55 | 168.45 |
| 48 | 2 | 5.76 | 81.08 | 1.97 | 76.67 | 17.45 | 202.26 | 114.36 |
| 49 | 2 | 5.11 | 95.83 | 7.03 | 94.12 | 10.77 | 206.04 | 109.48 |
| 50 | 2 | 7.69 | 181.48 | 10.12 | 173.64 | 9.89 | 241.82 | 125.74 |
| 51 | 2 | 11.45 | 164.52 | 22.63 | 174.04 | 26.92 | 262.57 | 152.71 |
| 52 | 2 | 6.67 | 98.23 | 3.35 | 93.64 | 4.00 | 132.48 | 67.47 |
| 53 | 2 | 21.77 | 157.36 | 12.91 | 165.38 | 31.88 | 292.41 | 171.98 |
| 54 | 2 | 12.06 | 232.61 | 15.70 | 226.85 | 35.19 | 292.27 | 175.21 |
| 55 | 2 | 5.06 | 232.25 | 17.34 | 223.22 | 26.20 | 324.11 | 181.48 |
| 56 | 2 | 12.13 | 228.81 | 13.66 | 221.55 | 18.82 | 281.06 | 153.48 |
| 57 | 2 | 8.30 | 190.49 | 21.61 | 193.41 | 21.27 | 312.35 | 170.92 |
| 58 | 2 | 26.44 | 167.85 | 24.18 | 189.09 | 39.45 | 345.36 | 204.92 |
| 59 | 2 | 3.34 | 221.84 | 29.52 | 225.12 | 44.52 | 206.25 | 143.34 |
| 60 | 2 | 7.54 | 165.09 | 5.75 | 154.89 | 2.66 | 178.09 | 87.98 |
| 61 | 2 | 9.08 | 163.76 | 10.18 | 159.25 | 25.80 | 249.63 | 145.40 |
| 62 | 2 | 16.66 | 182.74 | 17.91 | 188.87 | 20.34 | 416.18 | 219.73 |
| 63 | 2 | 13.16 | 218.67 | 21.26 | 221.04 | 10.04 | 159.73 | 86.57 |
| 64 | 2 | 9.67 | 266.84 | 36.23 | 275.50 | 205.05 | 155.02 | 279.32 |
| 65 | 2 | 5.70 | 164.87 | 16.78 | 164.41 | 148.59 | 150.43 | 220.66 |
| 66 | 2 | 4.47 | 121.92 | 7.03 | 116.39 | 24.19 | 205.99 | 122.88 |
| 67 | 2 | 3.39 | 213.72 | 32.19 | 220.75 | 9.96 | 158.41 | 85.85 |
| 68 | 2 | 36.31 | 158.45 | 58.22 | 221.92 | 19.37 | 385.96 | 204.28 |
| 69 | 2 | 6.62 | 197.64 | 9.42 | 186.26 | 34.18 | 216.56 | 137.93 |
| 70 | 2 | 12.08 | 140.76 | 41.89 | 173.05 | 33.03 | 350.20 | 200.81 |
| 71 | 2 | 33.07 | 266.31 | 25.85 | 281.22 | 3.23 | 435.66 | 211.95 |
| 72 | 2 | 9.09 | 161.82 | 11.97 | 159.35 | 18.16 | 249.83 | 137.85 |
| 73 | 2 | 7.94 | 223.78 | 9.48 | 210.02 | 106.08 | 263.08 | 232.12 |
| 74 | 2 | 7.98 | 204.67 | 14.84 | 198.76 | 33.66 | 270.96 | 163.48 |
| 75 | 2 | 4.05 | 157.69 | 13.94 | 154.16 | 7.18 | 118.70 | 64.05 |
| 76 | 2 | 24.79 | 96.62 | 13.53 | 115.22 | 11.58 | 245.64 | 129.27 |
| 77 | 2 | 6.10 | 170.41 | 17.43 | 170.17 | 29.56 | 190.52 | 120.84 |
| 78 | 2 | 20.83 | 169.13 | 19.31 | 181.36 | 18.72 | 229.57 | 128.71 |
| 79 | 2 | 13.11 | 100.36 | 34.60 | 131.30 | 3.97 | 130.00 | 66.25 |
| 80 | 2 | 5.87 | 174.96 | 5.87 | 162.42 | 10.13 | 99.96 | 58.02 |
| 81 | 2 | 13.10 | 176.10 | 8.16 | 170.82 | 30.10 | 227.89 | 139.28 |
| 82 | 2 | 28.62 | 258.68 | 23.36 | 268.92 | 17.46 | 263.17 | 143.55 |
| 83 | 2 | 6.71 | 202.31 | 22.16 | 203.12 | 43.82 | 189.57 | 134.64 |
| 84 | 2 | 10.88 | 139.85 | 11.93 | 141.44 | 6.85 | 152.31 | 79.83 |
| 85 | 2 | 5.62 | 179.00 | 18.50 | 178.39 | 8.99 | 115.12 | 64.14 |
| 86 | 2 | 20.86 | 160.00 | 14.25 | 168.37 | 32.54 | 233.68 | 144.49 |
| 87 | 2 | 16.09 | 91.55 | 17.39 | 108.51 | 11.31 | 411.54 | 208.48 |
| 88 | 2 | 34.95 | 205.16 | 25.72 | 229.14 | 1.19 | 46.24 | 23.34 |
| 89 | 2 | 11.41 | 245.52 | 38.28 | 260.21 | 26.11 | 226.67 | 134.71 |
| 90 | 2 | 14.39 | 270.14 | 35.97 | 281.45 | 37.35 | 200.24 | 133.28 |
| 91 | 2 | 9.17 | 101.93 | 8.49 | 103.76 | 3.46 | 125.18 | 63.43 |
| 92 | 2 | 5.34 | 220.05 | 10.13 | 205.59 | 4.04 | 128.28 | 65.50 |
| 93 | 2 | 8.51 | 201.72 | 11.44 | 193.17 | 62.07 | 264.73 | 188.90 |
| 94 | 2 | 14.73 | 101.89 | 10.37 | 109.54 | 0.52 | 102.75 | 49.75 |
| 95 | 2 | 19.35 | 216.06 | 26.51 | 228.40 | 11.05 | 252.69 | 132.12 |
| 96 | 2 | 8.06 | 208.49 | 18.01 | 205.32 | 9.90 | 162.89 | 87.94 |
| 97 | 2 | 7.86 | 158.56 | 7.80 | 151.48 | 12.01 | 133.66 | 76.05 |
| 98 | 2 | 7.98 | 183.22 | 3.42 | 168.66 | 19.01 | 163.67 | 97.43 |
| 99 | 2 | 4.74 | 189.19 | 16.39 | 184.53 | 18.35 | 123.39 | 77.46 |
| 100 | 2 | 5.40 | 74.26 | 2.07 | 70.58 | 3.44 | 131.10 | 66.25 |
| 101 | 2 | 9.91 | 80.09 | 20.41 | 97.18 | 4.63 | 187.03 | 94.23 |
| 102 | 2 | 5.52 | 36.15 | 2.13 | 37.52 | 90.72 | 196.75 | 184.99 |
| 103 | 2 | 7.66 | 86.78 | 20.29 | 101.30 | 12.27 | 209.69 | 112.73 |
| 104 | 2 | 11.06 | 249.00 | 15.89 | 240.61 | 80.41 | 266.37 | 208.03 |
| 105 | 2 | 21.81 | 189.25 | 24.33 | 204.60 | 5.88 | 171.70 | 88.14 |
| 106 | 2 | 11.64 | 101.16 | 3.23 | 99.59 | 2.69 | 200.31 | 98.66 |
| 107 | 2 | 6.43 | 228.42 | 8.87 | 212.38 | 22.21 | 241.96 | 138.13 |
| 108 | 2 | 6.18 | 169.36 | 7.64 | 159.53 | 7.35 | 138.68 | 73.79 |
| 109 | 2 | 13.71 | 148.22 | 12.58 | 151.38 | 45.16 | 253.29 | 166.51 |
| 110 | 2 | 13.53 | 225.60 | 30.69 | 236.77 | 34.09 | 152.98 | 107.38 |
| 111 | 2 | 3.78 | 138.25 | 3.87 | 126.97 | 33.67 | 186.43 | 122.99 |
| 112 | 2 | 6.60 | 175.91 | 13.28 | 171.18 | 52.14 | 126.73 | 112.86 |
| 113 | 2 | 7.98 | 155.86 | 4.09 | 145.50 | 1.99 | 161.80 | 79.51 |
| 114 | 2 | 4.85 | 107.67 | 12.34 | 109.56 | 5.46 | 101.27 | 53.98 |
| 115 | 2 | 12.51 | 178.61 | 14.03 | 178.46 | 13.31 | 418.93 | 214.02 |
| 116 | 2 | 13.06 | 68.17 | 28.94 | 97.56 | 27.23 | 144.80 | 96.60 |
| 117 | 2 | 8.11 | 211.23 | 10.60 | 200.33 | 2.45 | 152.66 | 75.59 |
| 118 | 2 | 5.34 | 140.50 | 3.03 | 129.19 | 4.50 | 99.75 | 52.29 |
| 119 | 2 | 9.99 | 182.04 | 17.51 | 183.13 | 39.16 | 310.26 | 187.81 |
| 120 | 2 | 9.87 | 178.63 | 32.04 | 194.62 | 547.74 | 439.51 | 758.31 |
| 121 | 2 | 11.45 | 217.92 | 9.46 | 207.38 | 31.35 | 684.45 | 359.27 |
| 122 | 2 | 17.43 | 201.42 | 21.42 | 209.20 | 14.76 | 449.34 | 230.04 |
| 123 | 2 | 7.60 | 87.67 | 9.28 | 91.02 | 2.47 | 231.34 | 113.30 |
| 124 | 2 | 11.06 | 177.92 | 31.98 | 194.78 | 63.24 | 180.04 | 149.50 |
| 125 | 2 | 8.56 | 141.28 | 5.08 | 134.20 | 88.45 | 233.17 | 200.17 |
| 126 | 2 | 7.75 | 132.09 | 14.83 | 135.37 | 159.90 | 174.08 | 243.30 |
| 127 | 2 | 12.25 | 166.68 | 13.66 | 167.51 | 8.61 | 221.92 | 114.93 |
| 128 | 2 | 6.65 | 153.07 | 3.76 | 141.80 | 4.73 | 176.68 | 89.38 |
| 129 | 2 | 11.95 | 142.35 | 10.56 | 143.00 | 1.08 | 112.24 | 54.85 |
| 130 | 2 | 28.16 | 144.11 | 20.80 | 166.25 | 13.55 | 262.19 | 139.16 |
| 131 | 2 | 8.92 | 141.23 | 7.90 | 137.23 | 7.55 | 142.38 | 75.77 |
| 132 | 2 | 4.66 | 122.10 | 3.35 | 113.00 | 45.06 | 128.57 | 106.66 |
| 133 | 2 | 14.69 | 205.64 | 30.43 | 219.94 | 9.99 | 210.65 | 110.91 |
| 134 | 2 | 7.56 | 143.52 | 3.50 | 133.86 | 13.25 | 312.56 | 163.00 |
| 135 | 2 | 6.95 | 165.19 | 10.39 | 159.20 | 3.58 | 126.31 | 64.09 |
| 136 | 2 | 15.04 | 188.71 | 20.28 | 195.30 | 22.19 | 183.67 | 110.19 |
| 137 | 2 | 6.36 | 167.91 | 11.12 | 161.87 | 10.15 | 235.83 | 123.13 |
| 138 | 2 | 6.47 | 71.55 | 17.65 | 84.56 | 8.65 | 103.39 | 58.18 |
| 139 | 2 | 16.05 | 59.87 | 7.72 | 71.23 | 27.10 | 217.52 | 131.31 |
| 140 | 2 | 6.14 | 188.61 | 5.10 | 173.73 | 42.82 | 217.50 | 147.03 |
| 141 | 6 | 5.10 | 245.51 | 41.61 | 259.07 | 11.27 | 85.68 | 52.32 |
| 142 | 6 | 4.89 | 339.27 | 25.78 | 324.76 | 17.03 | 143.69 | 85.87 |
| 143 | 6 | 3.03 | 162.93 | 22.96 | 167.02 | 194.10 | 213.29 | 296.29 |
| 144 | 6 | 7.97 | 224.32 | 15.92 | 216.96 | 9.47 | 112.37 | 63.30 |
| 145 | 6 | 7.46 | 303.95 | 38.77 | 308.80 | 14.83 | 149.16 | 86.30 |
| 146 | 6 | 5.14 | 211.85 | 34.42 | 222.58 | 4.69 | 140.76 | 72.13 |
| 147 | 6 | 2.32 | 220.36 | 9.15 | 202.73 | 6.53 | 221.37 | 112.59 |
| 148 | 6 | 7.37 | 183.49 | 36.15 | 201.20 | 5.43 | 164.75 | 84.36 |
| 149 | 6 | 5.73 | 240.25 | 13.62 | 226.94 | 14.75 | 164.09 | 93.36 |
| 150 | 6 | 6.65 | 207.94 | 16.81 | 202.64 | 15.15 | 210.98 | 116.23 |
| 151 | 6 | 5.22 | 265.47 | 23.60 | 258.53 | 21.29 | 165.62 | 100.64 |
| 152 | 6 | 8.64 | 200.34 | 47.46 | 228.07 | 8.81 | 283.28 | 144.53 |
| 153 | 6 | 6.02 | 293.84 | 11.43 | 271.63 | 2.53 | 127.22 | 63.48 |
| 154 | 6 | 5.50 | 276.84 | 8.25 | 253.28 | 11.37 | 157.91 | 87.03 |
| 155 | 6 | 6.92 | 27.60 | 11.12 | 40.06 | 6.11 | 141.93 | 74.11 |
| 156 | 6 | 5.70 | 99.98 | 121.75 | 212.87 | 5.54 | 168.33 | 86.19 |
| 157 | 6 | 8.73 | 184.18 | 33.44 | 200.04 | 178.72 | 405.60 | 373.05 |
| 158 | 6 | 5.25 | 195.84 | 20.38 | 194.68 | 123.46 | 171.25 | 205.50 |
| 159 | 6 | 14.31 | 271.68 | 34.36 | 281.13 | 43.14 | 564.29 | 313.49 |
| 160 | 6 | 5.63 | 265.08 | 35.36 | 270.25 | 290.09 | 197.82 | 384.86 |
| 161 | 6 | 3.76 | 250.37 | 11.93 | 232.67 | 25.07 | 275.17 | 156.91 |
| 162 | 6 | 8.06 | 233.37 | 26.16 | 235.13 | 23.17 | 179.66 | 109.25 |
| 163 | 6 | 62.60 | 56.82 | 18.91 | 112.68 | 20.88 | 259.88 | 145.39 |
| 164 | 6 | 4.84 | 252.79 | 40.54 | 264.16 | 34.90 | 182.02 | 122.11 |
| 165 | 6 | 6.90 | 272.79 | 29.53 | 272.02 | 15.18 | 178.48 | 100.69 |
| 166 | 6 | 3.80 | 275.08 | 15.88 | 258.18 | 11.57 | 193.38 | 104.22 |
| 167 | 6 | 5.78 | 254.38 | 23.73 | 249.40 | 24.42 | 159.80 | 100.98 |
| 168 | 6 | 8.19 | 254.70 | 59.83 | 287.48 | 29.90 | 322.21 | 184.27 |
| 169 | 6 | 10.34 | 95.18 | 11.64 | 101.86 | 4.43 | 76.50 | 41.08 |
| 170 | 6 | 23.21 | 142.96 | 36.67 | 177.61 | 14.71 | 199.93 | 110.50 |
| 171 | 6 | 6.98 | 204.60 | 11.13 | 194.28 | 3.13 | 173.22 | 86.12 |
| 172 | 6 | 4.82 | 184.80 | 23.42 | 187.80 | 63.76 | 167.95 | 144.23 |
| 173 | 6 | 7.54 | 223.41 | 18.81 | 218.75 | 41.00 | 197.26 | 135.51 |
| 174 | 6 | 13.31 | 180.56 | 9.97 | 176.66 | 0.42 | 99.82 | 48.24 |
| 175 | 6 | 8.41 | 278.85 | 22.33 | 271.17 | 8.28 | 148.14 | 79.25 |
| 176 | 6 | 0.77 | 17.47 | 0.01 | 15.77 | 5.95 | 136.41 | 71.31 |
| 177 | 6 | 4.40 | 179.60 | 11.41 | 170.97 | 7.23 | 109.45 | 59.67 |
| 178 | 6 | 13.17 | 342.28 | 28.55 | 336.01 | 165.86 | 292.77 | 306.12 |
| 179 | 6 | 4.52 | 172.30 | 39.40 | 192.68 | 13.95 | 215.06 | 116.98 |
| 180 | 6 | 2.14 | 184.36 | 40.84 | 202.95 | 13.95 | 133.72 | 78.02 |
| 181 | 6 | 11.41 | 83.87 | 22.08 | 103.21 | 20.22 | 346.83 | 186.39 |
| 182 | 6 | 6.85 | 200.96 | 12.07 | 191.96 | 109.66 | 246.80 | 227.90 |
| 183 | 6 | 8.90 | 188.45 | 30.18 | 200.63 | 51.04 | 326.97 | 207.69 |
| 184 | 6 | 3.94 | 102.83 | 13.87 | 106.22 | 9.08 | 310.83 | 158.00 |
| 185 | 6 | 9.15 | 170.34 | 36.63 | 191.48 | 26.45 | 318.60 | 179.09 |

TPP: thiamin pyrophosphate, TMP: thiamin monophosphate, FAD: flavin adenine dinucleotide
