# Supplementary material for: Thiamin and Riboflavin in Human Milk: Effects of Lipid-Based Nutrient Supplementation and Stage of Lactation on Vitamer Secretion and Contributions to Total Vitamin Content
Source: PLoS One. 2016 Feb 17;11(2):e0149479. doi: 10.1371/journal.pone.0149479 (PMC4757446; doi:10.1371/journal.pone.0149479)
Supplement: S3 Table — (DOCX) [file pone.0149479.s003.docx]

**S3 Table:** Concentrations of TPP, TMP, thiamin, total thiamin, riboflavin, FAD, and total riboflavin [µg/L] in the control group at 24 weeks.

| **Sample** | **TPP** | **TMP** | **thiamin** | **total thiamine** | **riboflavin** | **FAD** | **total riboflavin** |
| --- | --- | --- | --- | --- | --- | --- | --- |
| 1 | 1.82 | 119.25 | 42.75 | 147.92 | 0.42 | 65.48 | 31.79 |
| 2 | 4.64 | 188.56 | 37.58 | 205.10 | 5.19 | 232.05 | 116.37 |
| 3 | 6.26 | 122.94 | 63.99 | 175.51 | 9.23 | 305.66 | 155.67 |
| 4 | 4.21 | 182.82 | 42.32 | 204.54 | 1.93 | 112.76 | 55.95 |
| 5 | 17.06 | 107.68 | 90.87 | 196.73 | 4.32 | 142.18 | 72.44 |
| 6 | 1.36 | 105.58 | 81.70 | 174.63 | 5.28 | 130.51 | 67.81 |
| 7 | 2.59 | 183.78 | 17.28 | 179.19 | 2.91 | 137.57 | 68.82 |
| 8 | 7.62 | 109.48 | 113.23 | 213.98 | 3.49 | 149.63 | 75.18 |
| 9 | 2.18 | 177.94 | 46.40 | 202.93 | 3.61 | 144.75 | 72.96 |
| 10 | 6.33 | 210.06 | 42.65 | 230.09 | 5.19 | 184.66 | 93.66 |
| 11 | 7.67 | 200.11 | 64.51 | 244.24 | 3.66 | 205.70 | 102.21 |
| 12 | 9.27 | 235.05 | 91.33 | 302.63 | 0.74 | 189.83 | 91.69 |
| 13 | 9.93 | 107.04 | 97.49 | 197.75 | 12.61 | 196.26 | 106.64 |
| 14 | 1.47 | 187.08 | 25.45 | 189.45 | 18.39 | 123.80 | 77.70 |
| 15 | 6.73 | 209.56 | 50.78 | 238.08 | 17.68 | 239.92 | 132.63 |
| 16 | 3.37 | 224.77 | 13.96 | 212.14 | 7.18 | 151.67 | 79.85 |
| 17 | 11.56 | 136.29 | 69.15 | 196.04 | 5.80 | 243.26 | 122.34 |
| 18 | 4.14 | 57.64 | 187.06 | 240.18 | 11.25 | 232.94 | 122.85 |
| 19 | 13.06 | 85.25 | 59.48 | 142.98 | 3.74 | 359.36 | 175.91 |
| 20 | 10.19 | 107.29 | 42.42 | 143.08 | 8.40 | 302.15 | 153.16 |
| 21 | 9.13 | 153.29 | 33.83 | 173.81 | 21.07 | 240.93 | 136.50 |
| 22 | 1.26 | 62.80 | 8.95 | 64.55 | 2.51 | 158.30 | 78.35 |
| 23 | 14.80 | 306.10 | 56.02 | 333.12 | 12.57 | 288.25 | 150.67 |
| 24 | 32.27 | 99.24 | 134.95 | 244.21 | 5.95 | 264.69 | 132.76 |
| 25 | 2.56 | 131.76 | 27.13 | 143.72 | 5.92 | 132.60 | 69.45 |
| 26 | 9.99 | 131.88 | 19.63 | 141.57 | 13.82 | 197.70 | 108.53 |
| 27 | 1.70 | 171.25 | 14.84 | 165.21 | 11.56 | 178.83 | 97.24 |
| 28 | 6.73 | 95.94 | 188.06 | 276.38 | 5.88 | 139.18 | 72.55 |
| 29 | 2.32 | 126.60 | 28.93 | 140.85 | 8.91 | 112.72 | 62.91 |
| 30 | 6.38 | 22.87 | 107.38 | 131.81 | 12.72 | 339.18 | 175.22 |
| 31 | 7.46 | 236.33 | 24.78 | 235.92 | 6.00 | 252.30 | 126.88 |
| 32 | 3.20 | 37.23 | 90.02 | 124.72 | 3.54 | 129.20 | 65.44 |
| 33 | 17.31 | 58.73 | 31.89 | 95.29 | 8.47 | 233.32 | 120.26 |
| 34 | 7.70 | 14.63 | 178.13 | 196.32 | 5.74 | 153.44 | 79.25 |
| 35 | 12.11 | 211.97 | 39.93 | 233.14 | 4.72 | 218.06 | 109.19 |
| 36 | 6.25 | 127.74 | 15.68 | 131.37 | 6.55 | 124.15 | 66.03 |
| 37 | 5.44 | 188.70 | 44.69 | 212.91 | 10.63 | 225.74 | 118.78 |
| 38 | 11.12 | 212.34 | 36.01 | 228.84 | 2.61 | 162.70 | 80.55 |
| 39 | 3.96 | 163.52 | 6.28 | 151.51 | 11.07 | 249.80 | 130.75 |
| 40 | 11.41 | 235.79 | 58.03 | 271.49 | 10.76 | 131.76 | 73.88 |
| 41 | 1.50 | 2.53 | 77.45 | 80.71 | 4.25 | 166.64 | 84.09 |
| 42 | 2.06 | 123.64 | 106.14 | 215.29 | 7.74 | 57.35 | 35.21 |
| 43 | 7.79 | 287.12 | 50.49 | 306.10 | 8.16 | 194.18 | 101.20 |
| 44 | 1.75 | 141.73 | 50.55 | 175.24 | 6.21 | 112.90 | 60.30 |
| 45 | 2.81 | 171.97 | 72.11 | 223.89 | 18.90 | 169.46 | 100.08 |
| 46 | 10.32 | 113.06 | 66.93 | 172.71 | 8.64 | 123.84 | 67.97 |
| 47 | 3.89 | 163.85 | 24.91 | 170.38 | 9.69 | 64.23 | 40.47 |
| 48 | 3.12 | 103.79 | 39.59 | 132.20 | 11.35 | 135.17 | 76.10 |
| 49 | 7.73 | 236.60 | 34.66 | 246.21 | 7.60 | 139.92 | 74.63 |
| 50 | 8.34 | 21.83 | 100.42 | 125.34 | 21.93 | 253.46 | 143.36 |
| 51 | 2.72 | 175.64 | 75.26 | 230.18 | 12.84 | 243.69 | 129.59 |
| 52 | 6.45 | 150.95 | 46.96 | 183.01 | 41.87 | 248.96 | 161.14 |
| 53 | 4.29 | 241.81 | 37.42 | 251.08 | 6.26 | 201.13 | 102.63 |
| 54 | 5.47 | 241.11 | 18.18 | 232.08 | 2.63 | 196.54 | 96.80 |
| 55 | 8.17 | 193.75 | 13.51 | 188.06 | 13.70 | 150.98 | 86.03 |
| 56 | 0.75 | 70.99 | 15.56 | 77.92 | 3.30 | 75.50 | 39.47 |
| 57 | 12.10 | 86.11 | 68.05 | 151.61 | 10.09 | 489.21 | 244.47 |
| 58 | 9.02 | 133.50 | 115.78 | 238.45 | 15.75 | 284.53 | 152.07 |
| 59 | 3.10 | 198.80 | 40.04 | 215.40 | 6.97 | 341.47 | 170.57 |
| 60 | 5.69 | 207.23 | 48.94 | 233.47 | 5.33 | 178.70 | 90.95 |
| 61 | 3.11 | 213.49 | 42.07 | 230.23 | 5.81 | 156.92 | 80.99 |
| 62 | 9.49 | 266.00 | 29.58 | 267.99 | 11.16 | 357.48 | 182.43 |
| 63 | 27.43 | 188.74 | 149.66 | 333.46 | 14.90 | 363.54 | 189.08 |
| 64 | 10.37 | 22.86 | 113.63 | 140.88 | 0.82 | 179.95 | 87.04 |
| 65 | 6.19 | 218.03 | 22.19 | 216.48 | 7.20 | 201.27 | 103.63 |
| 66 | 12.07 | 136.64 | 121.46 | 249.02 | 21.00 | 260.17 | 145.65 |
| 67 | 3.02 | 218.89 | 13.71 | 206.50 | 5.99 | 246.63 | 124.15 |
| 68 | 7.59 | 188.71 | 23.97 | 193.71 | 7.85 | 243.09 | 124.32 |
| 69 | 26.82 | 254.54 | 39.21 | 279.89 | 11.20 | 565.42 | 282.10 |
| 70 | 2.36 | 187.10 | 25.09 | 189.73 | 5.84 | 134.16 | 70.11 |
| 71 | 6.04 | 204.80 | 101.19 | 283.86 | 12.47 | 224.57 | 120.06 |
| 72 | 6.54 | 210.80 | 25.76 | 214.01 | 7.39 | 208.17 | 107.12 |
| 73 | 6.92 | 195.83 | 30.33 | 205.81 | 5.61 | 166.50 | 85.38 |
| 74 | 3.41 | 71.88 | 56.96 | 121.99 | 1.79 | 126.96 | 62.62 |
| 75 | 6.56 | 153.31 | 22.34 | 160.51 | 4.50 | 165.73 | 83.90 |
| 76 | 11.39 | 92.16 | 18.17 | 106.50 | 15.95 | 217.09 | 119.96 |
| 77 | 5.78 | 205.41 | 79.54 | 262.55 | 11.13 | 134.02 | 75.34 |
| 78 | 1.13 | 119.46 | 36.71 | 141.57 | 6.45 | 132.15 | 69.77 |
| 79 | 2.50 | 176.55 | 25.42 | 180.97 | 3.91 | 149.32 | 75.45 |
| 80 | 3.74 | 68.20 | 26.52 | 88.57 | 10.26 | 279.98 | 144.40 |
| 81 | 5.52 | 234.65 | 29.13 | 237.43 | 2.51 | 152.00 | 75.33 |
| 82 | 8.17 | 137.32 | 134.67 | 260.06 | 8.27 | 206.18 | 107.05 |
| 83 | 3.11 | 178.37 | 32.41 | 189.98 | 10.41 | 228.58 | 119.92 |
| 84 | 4.75 | 169.04 | 41.95 | 192.56 | 12.56 | 311.22 | 161.67 |
| 85 | 10.06 | 353.25 | 38.34 | 353.15 | 10.22 | 177.55 | 95.28 |
| 86 | 13.09 | 197.60 | 42.24 | 223.61 | 12.95 | 264.30 | 139.58 |
| 87 | 5.55 | 83.80 | 85.47 | 162.39 | 0.48 | 69.82 | 33.93 |
| 88 | 6.07 | 215.45 | 24.38 | 216.34 | 30.93 | 187.87 | 120.94 |
| 89 | 11.57 | 284.71 | 17.15 | 273.33 | 9.31 | 258.84 | 133.32 |
| 90 | 34.00 | 170.01 | 61.33 | 233.47 | 19.73 | 299.19 | 163.07 |
| 91 | 6.43 | 200.61 | 10.27 | 189.56 | 5.60 | 181.66 | 92.64 |
| 92 | 6.21 | 188.76 | 18.30 | 187.11 | 5.43 | 262.28 | 131.09 |
| 93 | 4.06 | 58.84 | 122.83 | 176.96 | 15.91 | 216.80 | 119.78 |
| 94 | 26.17 | 40.07 | 21.42 | 74.84 | 12.90 | 366.21 | 188.36 |
| 95 | 6.47 | 253.89 | 16.79 | 242.52 | 3.79 | 171.02 | 85.72 |
| 96 | 17.36 | 211.76 | 23.74 | 220.47 | 4.21 | 206.73 | 103.25 |
| 97 | 4.40 | 144.48 | 29.11 | 158.07 | 1.25 | 136.11 | 66.46 |
| 98 | 3.76 | 164.23 | 21.83 | 167.54 | 0.96 | 130.06 | 63.27 |
| 99 | 7.05 | 200.35 | 27.40 | 206.90 | 6.28 | 188.71 | 96.69 |
| 100 | 9.95 | 178.04 | 41.23 | 203.36 | 19.13 | 506.75 | 261.91 |
| 101 | 8.73 | 158.42 | 33.85 | 178.02 | 0.80 | 50.30 | 24.90 |
| 102 | 12.56 | 249.39 | 29.98 | 256.09 | 1.23 | 82.93 | 40.97 |
| 103 | 10.71 | 140.75 | 29.35 | 159.53 | 20.67 | 127.03 | 81.53 |
| 104 | 10.97 | 151.63 | 37.59 | 177.43 | 8.75 | 178.29 | 94.17 |
| 105 | 3.91 | 102.02 | 10.51 | 102.14 | 0.70 | 66.35 | 32.49 |
| 106 | 0.94 | 264.84 | 78.76 | 310.11 | 6.01 | 139.20 | 72.70 |
| 107 | 2.99 | 199.21 | 30.47 | 206.11 | 0.77 | 50.36 | 24.90 |
| 108 | 6.31 | 182.01 | 35.63 | 198.63 | 4.68 | 175.54 | 88.78 |
| 109 | 13.29 | 227.05 | 30.82 | 237.99 | 4.31 | 40.98 | 23.94 |
| 110 | 6.79 | 75.78 | 23.49 | 94.30 | 0.13 | 38.21 | 18.44 |
| 111 | 14.27 | 150.36 | 44.31 | 185.37 | 4.43 | 284.53 | 140.75 |
| 112 | 12.39 | 230.20 | 118.56 | 327.84 | 3.12 | 59.68 | 31.71 |
| 113 | 3.31 | 202.31 | 22.70 | 201.27 | 5.34 | 164.00 | 83.92 |
| 114 | 12.64 | 88.77 | 79.36 | 165.63 | 10.19 | 254.68 | 132.21 |
| 115 | 1.98 | 204.31 | 48.42 | 227.79 | 35.02 | 162.84 | 113.04 |
| 116 | 5.77 | 285.45 | 34.51 | 287.23 | 11.48 | 184.42 | 99.84 |
| 117 | 5.43 | 201.00 | 13.77 | 192.69 | 1.29 | 91.33 | 45.04 |
| 118 | 5.36 | 157.68 | 20.79 | 161.93 | 1.31 | 159.12 | 77.54 |
| 119 | 15.88 | 217.42 | 10.92 | 211.54 | 6.00 | 82.60 | 45.57 |
| 120 | 4.82 | 74.93 | 25.25 | 93.93 | 0.27 | 42.79 | 20.78 |
| 121 | 6.50 | 146.91 | 36.62 | 169.18 | 7.43 | 211.52 | 108.77 |
| 122 | 5.43 | 196.10 | 53.58 | 228.23 | 8.65 | 87.13 | 50.39 |
| 123 | 6.12 | 201.69 | 28.41 | 208.42 | 2.20 | 68.29 | 34.92 |
| 124 | 2.89 | 93.45 | 45.92 | 129.36 | 13.87 | 177.21 | 98.77 |
| 125 | 8.37 | 230.98 | 26.37 | 233.49 | 10.52 | 222.70 | 117.21 |
| 126 | 12.84 | 262.07 | 49.43 | 286.79 | 17.55 | 197.75 | 112.29 |
| 127 | 5.27 | 141.42 | 56.34 | 183.25 | 3.18 | 104.87 | 53.42 |
| 128 | 4.58 | 184.30 | 13.89 | 177.67 | 3.97 | 353.20 | 173.19 |
| 129 | 3.09 | 78.11 | 50.61 | 120.83 | 2.06 | 80.48 | 40.62 |
| 130 | 21.88 | 120.37 | 127.62 | 247.95 | 13.25 | 123.34 | 72.34 |
| 131 | 3.36 | 176.36 | 77.20 | 233.20 | 27.95 | 94.93 | 73.43 |
| 132 | 8.11 | 178.29 | 27.01 | 188.04 | 2.23 | 86.55 | 43.69 |
| 133 | 3.54 | 259.42 | 40.74 | 269.22 | 23.75 | 153.79 | 97.43 |
| 134 | 3.52 | 350.57 | 22.03 | 329.88 | 3.78 | 60.24 | 32.64 |
| 135 | 0.63 | 12.24 | 3.23 | 14.33 | 1.62 | 42.87 | 22.16 |
| 136 | 3.67 | 103.88 | 102.03 | 195.11 | 2.94 | 54.23 | 28.92 |
| 137 | 6.08 | 129.53 | 40.50 | 157.63 | 5.19 | 177.76 | 90.36 |
| 138 | 13.84 | 128.75 | 147.51 | 269.45 | 4.19 | 90.09 | 47.35 |
| 139 | 8.48 | 101.23 | 15.90 | 110.07 | 10.78 | 180.62 | 97.31 |
| 140 | 20.29 | 244.30 | 40.38 | 267.53 | 9.96 | 165.06 | 89.04 |
| 141 | 11.21 | 90.17 | 66.63 | 153.09 | 9.02 | 209.21 | 109.25 |
| 142 | 6.55 | 234.16 | 40.12 | 248.72 | 3.09 | 155.21 | 77.45 |
| 143 | 6.59 | 262.46 | 36.29 | 269.57 | 7.00 | 186.77 | 96.48 |
| 144 | 3.31 | 144.69 | 48.56 | 176.93 | 5.92 | 251.51 | 126.42 |
| 145 | 9.66 | 43.87 | 74.19 | 119.24 | 81.39 | 673.70 | 404.17 |
| 146 | 1.42 | 83.80 | 7.24 | 81.24 | 3.79 | 50.54 | 28.01 |
| 147 | 9.88 | 132.25 | 58.67 | 180.86 | 4.95 | 245.09 | 122.37 |
| 148 | 1.34 | 136.48 | 91.81 | 211.64 | 10.76 | 86.27 | 52.09 |
| 149 | 1.82 | 85.68 | 35.16 | 111.08 | 6.12 | 80.15 | 44.52 |
| 150 | 4.57 | 69.93 | 21.65 | 85.80 | 22.90 | 179.27 | 108.79 |
| 151 | 4.21 | 219.45 | 79.94 | 274.06 | 2.27 | 66.06 | 33.92 |
| 152 | 15.79 | 143.09 | 23.60 | 159.41 | 1.81 | 97.49 | 48.52 |
| 153 | 2.61 | 92.32 | 109.47 | 191.74 | 223.13 | 136.28 | 288.42 |
| 154 | 15.17 | 206.63 | 57.52 | 248.23 | 20.69 | 197.68 | 115.40 |
| 155 | 11.42 | 289.20 | 44.82 | 304.80 | 4.12 | 179.83 | 90.28 |
| 156 | 5.95 | 190.11 | 76.31 | 246.11 | 1.67 | 100.81 | 49.97 |
| 157 | 21.78 | 186.45 | 56.95 | 234.76 | 23.88 | 211.80 | 125.35 |
| 158 | 19.92 | 128.72 | 23.99 | 150.20 | 18.92 | 360.35 | 191.56 |
| 159 | 23.41 | 90.19 | 95.92 | 191.05 | 10.61 | 207.35 | 109.95 |
| 160 | 35.37 | 290.22 | 68.76 | 346.57 | 11.67 | 432.44 | 218.85 |
| 161 | 4.28 | 105.47 | 12.58 | 107.48 | 0.76 | 52.80 | 26.06 |
| 162 | 3.76 | 184.01 | 60.55 | 223.50 | 15.18 | 212.23 | 116.87 |
| 163 | 5.09 | 113.18 | 6.59 | 108.78 | 7.76 | 203.44 | 105.22 |
| 164 | 4.44 | 69.18 | 84.91 | 148.31 | 7.77 | 56.06 | 34.63 |
| 165 | 4.75 | 88.71 | 8.41 | 89.04 | 15.21 | 125.42 | 75.29 |
| 166 | 2.95 | 222.30 | 45.46 | 241.18 | 13.78 | 72.23 | 48.39 |
| 167 | 10.99 | 269.82 | 28.21 | 271.00 | 4.11 | 135.70 | 69.12 |
| 168 | 10.55 | 166.60 | 54.70 | 207.28 | 4.54 | 136.32 | 69.86 |
| 169 | 10.34 | 267.85 | 33.92 | 274.55 | 7.41 | 337.16 | 168.95 |
| 170 | 8.01 | 118.97 | 45.48 | 154.78 | 13.43 | 156.90 | 88.60 |
| 171 | 4.81 | 119.25 | 158.42 | 265.70 | 19.59 | 163.76 | 98.05 |
| 172 | 9.80 | 146.14 | 27.73 | 161.96 | 26.46 | 282.82 | 161.96 |
| 173 | 9.20 | 18.61 | 224.32 | 247.04 | 2.84 | 292.97 | 143.20 |
| 174 | 10.93 | 176.34 | 53.83 | 215.17 | 33.33 | 416.78 | 233.01 |
| 175 | 4.09 | 185.83 | 25.83 | 190.59 | 1.21 | 144.93 | 70.65 |
| 176 | 6.21 | 244.15 | 40.88 | 257.94 | 8.29 | 266.92 | 136.18 |
| 177 | 8.66 | 130.02 | 83.48 | 202.86 | 52.20 | 517.16 | 299.97 |

TPP: thiamin pyrophosphate, TMP: thiamin monophosphate, FAD: flavin adenine dinucleotide
