# Supplementary material for: Thiamin and Riboflavin in Human Milk: Effects of Lipid-Based Nutrient Supplementation and Stage of Lactation on Vitamer Secretion and Contributions to Total Vitamin Content
Source: PLoS One. 2016 Feb 17;11(2):e0149479. doi: 10.1371/journal.pone.0149479 (PMC4757446; doi:10.1371/journal.pone.0149479)
Supplement: S4 Table — (DOCX) [file pone.0149479.s004.docx]

**S4 Table:** Concentrations of TPP, TMP, thiamin, total thiamin, riboflavin, FAD, and total riboflavin [µg/L] in the LNS group at 24 weeks.

| **Sample** | **TPP** | **TMP** | **thiamin** | **total thiamin** | **riboflavin** | **FAD** | **total riboflavin** |
| --- | --- | --- | --- | --- | --- | --- | --- |
| 1 | 9.45 | 86.31 | 165.62 | 247.49 | 52.26 | 327.50 | 209.16 |
| 2 | 6.15 | 134.00 | 105.96 | 227.04 | 12.51 | 86.37 | 53.89 |
| 3 | 1.86 | 99.47 | 54.12 | 142.08 | 6.75 | 132.56 | 70.26 |
| 4 | 3.00 | 220.40 | 52.43 | 246.54 | 17.19 | 86.81 | 58.78 |
| 5 | 6.33 | 249.97 | 26.75 | 248.96 | 19.50 | 288.11 | 157.53 |
| 6 | 7.98 | 94.90 | 71.77 | 160.08 | 9.35 | 303.64 | 154.83 |
| 7 | 15.06 | 240.71 | 74.95 | 295.27 | 314.53 | 406.97 | 509.51 |
| 8 | 19.33 | 6.62 | 188.12 | 207.55 | 34.59 | 201.14 | 130.96 |
| 9 | 7.62 | 257.34 | 63.26 | 292.80 | 98.46 | 198.38 | 193.50 |
| 10 | 9.94 | 227.33 | 61.08 | 266.13 | 20.59 | 555.27 | 286.62 |
| 11 | 24.69 | 146.43 | 23.83 | 168.84 | 24.00 | 280.10 | 158.20 |
| 12 | 13.24 | 113.96 | 39.95 | 148.58 | 22.48 | 227.90 | 131.66 |
| 13 | 6.69 | 23.26 | 170.81 | 195.80 | 18.14 | 173.99 | 101.50 |
| 14 | 4.39 | 174.60 | 40.43 | 195.62 | 34.62 | 196.61 | 128.82 |
| 15 | 9.74 | 95.33 | 5.32 | 95.25 | 5.42 | 226.70 | 114.03 |
| 16 | 9.26 | 152.10 | 54.23 | 193.27 | 81.53 | 370.59 | 259.08 |
| 17 | 1.42 | 191.88 | 21.96 | 190.10 | 6.64 | 140.49 | 73.95 |
| 18 | 4.83 | 234.08 | 27.77 | 235.09 | 10.15 | 201.30 | 106.59 |
| 19 | 10.46 | 122.28 | 40.44 | 154.36 | 21.38 | 258.72 | 145.33 |
| 20 | 5.36 | 212.32 | 27.72 | 216.46 | 4.92 | 278.01 | 138.11 |
| 21 | 12.96 | 73.29 | 135.69 | 208.70 | 2.91 | 304.32 | 148.71 |
| 22 | 28.61 | 181.22 | 22.61 | 200.69 | 28.76 | 287.36 | 166.43 |
| 23 | 10.59 | 123.82 | 59.68 | 175.02 | 4.61 | 270.60 | 134.25 |
| 24 | 6.49 | 214.10 | 32.34 | 223.43 | 3.22 | 247.06 | 121.59 |
| 25 | 3.90 | 289.19 | 44.53 | 299.19 | 43.80 | 178.74 | 129.43 |
| 26 | 3.04 | 180.99 | 20.71 | 180.51 | 5.48 | 75.55 | 41.68 |
| 27 | 1.92 | 76.78 | 88.81 | 157.04 | 20.51 | 129.18 | 82.40 |
| 28 | 11.01 | 233.52 | 14.20 | 225.39 | 17.11 | 233.84 | 129.14 |
| 29 | 5.56 | 217.94 | 16.36 | 210.13 | 12.83 | 90.83 | 56.35 |
| 30 | 4.99 | 95.07 | 105.73 | 192.07 | 7.63 | 324.47 | 163.08 |
| 31 | 7.78 | 239.61 | 38.50 | 252.72 | 2.98 | 114.02 | 57.61 |
| 32 | 8.14 | 119.30 | 75.58 | 185.26 | 11.12 | 313.07 | 161.11 |
| 33 | 5.47 | 143.10 | 110.08 | 238.60 | 18.18 | 485.46 | 250.76 |
| 34 | 6.49 | 219.73 | 41.52 | 237.51 | 53.29 | 165.54 | 132.60 |
| 35 | 4.00 | 198.21 | 77.05 | 252.54 | 94.42 | 249.22 | 213.82 |
| 36 | 3.93 | 153.90 | 37.37 | 174.20 | 13.16 | 133.25 | 77.00 |
| 37 | 4.33 | 156.95 | 55.20 | 194.97 | 56.12 | 204.68 | 154.18 |
| 38 | 16.35 | 174.12 | 68.07 | 231.30 | 43.81 | 156.51 | 118.80 |
| 39 | 3.93 | 164.49 | 56.13 | 202.19 | 44.99 | 137.49 | 110.86 |
| 40 | 9.77 | 78.50 | 131.46 | 206.75 | 78.90 | 482.00 | 309.82 |
| 41 | 8.62 | 314.03 | 61.66 | 341.30 | 110.24 | 330.19 | 268.43 |
| 42 | 3.57 | 168.73 | 79.60 | 229.09 | 29.85 | 319.03 | 182.70 |
| 43 | 13.67 | 153.31 | 80.09 | 223.29 | 15.61 | 398.12 | 206.35 |
| 44 | 9.68 | 245.64 | 61.32 | 282.13 | 178.67 | 195.20 | 272.19 |
| 45 | 2.28 | 240.96 | 8.74 | 220.24 | 20.21 | 152.08 | 93.08 |
| 46 | 2.88 | 28.34 | 97.22 | 123.94 | 11.41 | 204.35 | 109.32 |
| 47 | 29.15 | 247.01 | 67.58 | 303.35 | 22.02 | 379.37 | 203.77 |
| 48 | 11.01 | 145.56 | 66.40 | 200.97 | 2.43 | 227.98 | 111.66 |
| 49 | 1.41 | 80.07 | 122.98 | 193.72 | 9.47 | 269.59 | 138.64 |
| 50 | 12.17 | 19.45 | 171.17 | 196.72 | 126.04 | 240.71 | 241.37 |
| 51 | 8.98 | 214.29 | 33.66 | 226.67 | 21.86 | 290.63 | 161.10 |
| 52 | 16.08 | 190.85 | 67.94 | 245.56 | 23.85 | 474.35 | 251.11 |
| 53 | 5.21 | 3.69 | 82.75 | 89.65 | 9.57 | 273.22 | 140.47 |
| 54 | 3.49 | 143.13 | 43.04 | 170.19 | 51.48 | 286.88 | 188.93 |
| 55 | 15.44 | 188.21 | 47.37 | 222.23 | 52.88 | 519.06 | 301.56 |
| 56 | 3.14 | 262.51 | 56.65 | 287.52 | 19.02 | 391.49 | 206.58 |
| 57 | 10.71 | 187.45 | 53.02 | 223.87 | 87.70 | 352.07 | 256.38 |
| 58 | 12.23 | 167.93 | 100.09 | 255.01 | 42.48 | 187.67 | 132.39 |
| 59 | 5.64 | 192.78 | 59.90 | 231.81 | 138.71 | 269.78 | 267.96 |
| 60 | 10.77 | 150.25 | 136.38 | 274.87 | 152.59 | 270.17 | 282.03 |
| 61 | 4.41 | 138.86 | 45.16 | 169.23 | 4.96 | 261.18 | 130.09 |
| 62 | 0.98 | 133.80 | 37.35 | 154.59 | 2.69 | 71.38 | 36.89 |
| 63 | 4.90 | 103.41 | 64.27 | 157.81 | 12.26 | 262.31 | 137.93 |
| 64 | 9.17 | 249.02 | 53.32 | 276.72 | 36.60 | 451.65 | 252.98 |
| 65 | 2.89 | 175.14 | 23.43 | 178.03 | 50.97 | 234.41 | 163.28 |
| 66 | 4.23 | 169.75 | 31.37 | 182.23 | 1.09 | 183.72 | 89.11 |
| 67 | 79.12 | 88.57 | 20.59 | 153.70 | 14.79 | 579.46 | 292.41 |
| 68 | 17.06 | 239.84 | 47.93 | 268.90 | 39.73 | 401.35 | 232.02 |
| 69 | 5.07 | 197.18 | 38.91 | 214.24 | 18.08 | 380.76 | 200.50 |
| 70 | 4.43 | 74.94 | 101.88 | 170.29 | 21.38 | 200.19 | 117.29 |
| 71 | 1.81 | 180.40 | 36.45 | 194.86 | 10.13 | 339.74 | 172.90 |
| 72 | 3.85 | 188.53 | 71.81 | 238.75 | 29.98 | 171.86 | 112.32 |
| 73 | 15.20 | 41.83 | 146.20 | 193.39 | 126.21 | 297.68 | 268.83 |
| 74 | 4.31 | 127.52 | 83.77 | 197.90 | 6.03 | 71.76 | 40.41 |
| 75 | 14.82 | 43.98 | 22.39 | 71.18 | 9.18 | 146.83 | 79.52 |
| 76 | 4.85 | 162.56 | 35.75 | 180.78 | 18.66 | 156.77 | 93.77 |
| 77 | 7.39 | 235.09 | 34.63 | 244.62 | 16.48 | 281.69 | 151.43 |
| 78 | 14.17 | 210.81 | 70.32 | 263.97 | 16.20 | 254.78 | 138.26 |
| 79 | 10.20 | 155.97 | 68.01 | 211.08 | 30.24 | 202.95 | 127.48 |
| 80 | 5.12 | 126.64 | 50.72 | 164.65 | 1.48 | 184.72 | 89.98 |
| 81 | 4.03 | 200.16 | 29.69 | 206.89 | 9.96 | 217.72 | 114.27 |
| 82 | 7.09 | 280.75 | 53.83 | 303.40 | 84.32 | 257.04 | 207.47 |
| 83 | 3.00 | 58.70 | 8.77 | 62.02 | 3.58 | 203.33 | 101.00 |
| 84 | 13.57 | 199.09 | 25.48 | 208.49 | 25.91 | 274.57 | 157.46 |
| 85 | 7.44 | 84.20 | 57.60 | 136.21 | 8.86 | 208.82 | 108.90 |
| 86 | 2.70 | 126.32 | 43.18 | 155.12 | 16.10 | 157.30 | 91.46 |
| 87 | 3.81 | 230.66 | 31.42 | 235.03 | 22.32 | 129.02 | 84.13 |
| 88 | 5.03 | 149.49 | 81.95 | 215.72 | 10.67 | 168.20 | 91.25 |
| 89 | 9.41 | 195.54 | 140.54 | 317.52 | 26.17 | 216.60 | 129.94 |
| 90 | 7.76 | 206.04 | 35.80 | 220.76 | 36.10 | 126.12 | 96.52 |
| 91 | 3.78 | 137.98 | 12.00 | 134.87 | 16.05 | 164.49 | 94.86 |
| 92 | 4.51 | 23.49 | 39.90 | 63.55 | 13.73 | 236.36 | 126.97 |
| 93 | 8.88 | 172.24 | 96.95 | 253.26 | 15.15 | 141.93 | 83.15 |
| 94 | 5.26 | 207.28 | 54.48 | 238.75 | 26.32 | 184.91 | 114.91 |
| 95 | 6.23 | 217.26 | 28.43 | 222.08 | 28.24 | 318.84 | 181.00 |
| 96 | 6.28 | 112.13 | 66.57 | 168.69 | 24.21 | 313.48 | 174.40 |
| 97 | 6.32 | 238.19 | 14.91 | 226.86 | 9.98 | 148.80 | 81.27 |
| 98 | 3.38 | 177.98 | 30.42 | 187.85 | 4.06 | 77.53 | 41.20 |
| 99 | 4.48 | 223.04 | 21.72 | 219.18 | 7.33 | 164.29 | 86.04 |
| 100 | 7.10 | 202.78 | 23.15 | 204.80 | 16.49 | 272.96 | 147.27 |
| 101 | 8.13 | 169.84 | 65.85 | 219.53 | 207.01 | 216.20 | 310.59 |
| 102 | 4.59 | 218.39 | 33.86 | 227.34 | 12.54 | 261.68 | 137.91 |
| 103 | 8.39 | 152.22 | 15.14 | 153.66 | 7.68 | 97.59 | 54.43 |
| 104 | 12.00 | 162.82 | 49.18 | 199.49 | 3.44 | 149.26 | 74.94 |
| 105 | 10.22 | 197.08 | 35.49 | 214.39 | 7.83 | 242.46 | 123.99 |
| 106 | 8.18 | 15.81 | 28.18 | 47.73 | 23.90 | 155.89 | 98.59 |
| 107 | 7.45 | 268.03 | 20.92 | 259.66 | 21.40 | 231.76 | 132.44 |
| 108 | 5.82 | 193.13 | 24.48 | 196.82 | 1.64 | 84.16 | 41.96 |
| 109 | 5.01 | 183.06 | 42.75 | 205.75 | 35.16 | 291.79 | 174.95 |
| 110 | 5.18 | 62.68 | 19.06 | 77.32 | 7.79 | 101.12 | 56.24 |
| 111 | 2.47 | 210.03 | 18.19 | 202.89 | 12.83 | 115.08 | 67.97 |
| 112 | 9.49 | 60.61 | 97.88 | 157.39 | 32.76 | 173.95 | 116.10 |
| 113 | 3.85 | 318.06 | 19.79 | 299.56 | 14.39 | 78.74 | 52.11 |
| 114 | 3.42 | 234.19 | 35.94 | 242.34 | 28.30 | 176.32 | 112.77 |
| 115 | 6.63 | 57.24 | 36.52 | 91.06 | 1.80 | 154.56 | 75.85 |
| 116 | 5.80 | 73.66 | 86.31 | 154.58 | 21.37 | 110.50 | 74.30 |
| 117 | 35.21 | 150.24 | 85.23 | 241.01 | 26.95 | 296.45 | 168.98 |
| 118 | 34.48 | 153.34 | 56.54 | 214.49 | 25.05 | 269.52 | 154.18 |
| 119 | 12.55 | 169.52 | 69.91 | 226.45 | 6.44 | 178.14 | 91.79 |
| 120 | 21.09 | 281.01 | 58.37 | 318.06 | 51.00 | 350.37 | 218.86 |
| 121 | 4.26 | 265.18 | 28.77 | 262.77 | 19.75 | 173.99 | 103.11 |
| 122 | 5.98 | 181.88 | 65.95 | 228.60 | 11.93 | 109.47 | 64.38 |
| 123 | 7.97 | 164.14 | 20.36 | 168.97 | 1.31 | 134.47 | 65.74 |
| 124 | 6.34 | 5.34 | 13.47 | 22.61 | 71.26 | 111.14 | 124.51 |
| 125 | 11.00 | 331.97 | 24.90 | 321.85 | 56.74 | 281.50 | 191.60 |
| 126 | 7.34 | 144.55 | 42.09 | 173.19 | 1.54 | 124.64 | 61.25 |
| 127 | 14.43 | 214.37 | 51.65 | 248.59 | 82.20 | 484.96 | 314.54 |
| 128 | 8.94 | 48.87 | 190.41 | 239.30 | 12.65 | 138.87 | 79.18 |
| 129 | 9.66 | 214.48 | 55.47 | 249.13 | 28.61 | 190.96 | 120.10 |
| 130 | 13.30 | 120.71 | 112.22 | 226.77 | 21.77 | 299.05 | 165.05 |
| 131 | 3.29 | 296.24 | 26.87 | 287.24 | 28.90 | 114.89 | 83.94 |
| 132 | 1.96 | 237.50 | 21.52 | 229.78 | 1.91 | 40.41 | 21.27 |
| 133 | 9.67 | 21.20 | 79.79 | 105.10 | 23.56 | 191.56 | 115.34 |
| 134 | 3.47 | 128.39 | 9.98 | 124.28 | 11.47 | 90.90 | 55.02 |
| 135 | 3.73 | 130.53 | 17.14 | 133.47 | 11.62 | 116.42 | 67.40 |
| 136 | 5.57 | 118.58 | 84.37 | 191.60 | 189.42 | 273.92 | 320.66 |
| 137 | 6.75 | 261.47 | 21.98 | 254.51 | 53.32 | 106.41 | 104.30 |
| 138 | 1.06 | 283.76 | 18.75 | 266.67 | 6.69 | 116.98 | 62.74 |
| 139 | 5.29 | 201.15 | 17.69 | 196.65 | 7.00 | 163.94 | 85.54 |
| 140 | 3.30 | 137.01 | 50.59 | 172.27 | 6.15 | 98.64 | 53.41 |
| 141 | 3.40 | 145.50 | 61.33 | 190.48 | 74.44 | 118.49 | 131.21 |
| 142 | 18.52 | 140.23 | 52.41 | 187.66 | 26.54 | 331.10 | 185.17 |
| 143 | 4.16 | 229.19 | 68.10 | 270.69 | 24.16 | 104.90 | 74.41 |
| 144 | 13.36 | 166.68 | 20.11 | 174.75 | 1.88 | 94.53 | 47.16 |
| 145 | 3.65 | 84.53 | 98.10 | 174.31 | 21.08 | 130.96 | 83.83 |
| 146 | 22.50 | 201.95 | 51.75 | 243.57 | 66.25 | 190.57 | 157.55 |
| 147 | 7.76 | 203.16 | 44.81 | 227.25 | 4.23 | 220.36 | 109.81 |
| 148 | 7.62 | 164.05 | 19.62 | 167.91 | 0.75 | 122.33 | 59.36 |
| 149 | 2.23 | 196.86 | 30.44 | 203.50 | 41.98 | 178.55 | 127.52 |
| 150 | 10.69 | 190.71 | 59.48 | 233.16 | 12.46 | 174.86 | 96.24 |
| 151 | 3.60 | 160.06 | 55.24 | 197.21 | 10.85 | 160.75 | 87.86 |
| 152 | 13.80 | 115.05 | 84.16 | 194.13 | 19.42 | 140.82 | 86.89 |
| 153 | 13.47 | 240.49 | 30.39 | 249.40 | 11.03 | 385.45 | 195.70 |
| 154 | 3.85 | 119.46 | 32.00 | 138.79 | 746.90 | 246.82 | 865.15 |
| 155 | 7.12 | 214.98 | 25.43 | 217.72 | 29.39 | 212.61 | 131.25 |
| 156 | 6.76 | 172.31 | 27.39 | 182.25 | 43.78 | 317.69 | 195.98 |
| 157 | 1.08 | 43.51 | 53.66 | 92.32 | 2.74 | 177.87 | 87.95 |
| 158 | 3.76 | 91.65 | 15.85 | 98.34 | 18.45 | 88.24 | 60.73 |
| 159 | 5.09 | 77.83 | 23.70 | 95.10 | 26.69 | 175.63 | 110.84 |
| 160 | 3.37 | 68.05 | 43.19 | 104.85 | 33.36 | 130.62 | 95.94 |
| 161 | 6.15 | 155.78 | 61.25 | 201.29 | 15.52 | 259.64 | 139.91 |
| 162 | 10.53 | 214.98 | 71.90 | 266.61 | 63.37 | 138.67 | 129.80 |
| 163 | 7.28 | 183.01 | 101.47 | 266.03 | 165.39 | 158.01 | 241.10 |
| 164 | 20.84 | 192.23 | 41.33 | 223.52 | 17.97 | 272.29 | 148.42 |
| 165 | 5.50 | 180.66 | 70.87 | 232.13 | 7.01 | 83.91 | 47.22 |
| 166 | 10.06 | 269.21 | 76.97 | 318.58 | 294.25 | 151.39 | 366.78 |
| 167 | 3.80 | 125.15 | 116.59 | 228.29 | 40.89 | 138.65 | 107.32 |
| 168 | 4.33 | 136.38 | 25.95 | 147.80 | 29.15 | 308.14 | 176.78 |
| 169 | 10.56 | 107.73 | 88.58 | 189.88 | 10.18 | 324.01 | 165.41 |
| 170 | 6.99 | 286.64 | 30.02 | 284.65 | 8.51 | 170.02 | 89.97 |
| 171 | 8.29 | 190.25 | 90.83 | 262.41 | 14.14 | 283.23 | 149.83 |
| 172 | 9.74 | 241.70 | 27.70 | 245.11 | 47.52 | 168.26 | 128.14 |
| 173 | 7.46 | 201.84 | 28.11 | 209.20 | 16.72 | 238.80 | 131.13 |
| 174 | 7.56 | 228.59 | 45.81 | 250.27 | 8.71 | 227.02 | 117.48 |
| 175 | 2.62 | 174.70 | 41.91 | 195.94 | 16.12 | 100.30 | 64.18 |
| 176 | 4.29 | 84.27 | 103.92 | 180.36 | 52.22 | 179.80 | 138.37 |
| 177 | 3.62 | 225.31 | 20.65 | 219.47 | 144.04 | 143.59 | 212.83 |
| 178 | 2.12 | 122.29 | 59.76 | 167.78 | 21.70 | 197.03 | 116.10 |
| 179 | 4.99 | 111.37 | 60.94 | 161.48 | 11.43 | 112.72 | 65.43 |
| 180 | 4.45 | 71.31 | 35.71 | 100.97 | 15.20 | 243.21 | 131.72 |
| 181 | 11.52 | 253.87 | 35.47 | 264.75 | 113.25 | 215.08 | 216.29 |
| 182 | 7.77 | 264.64 | 24.93 | 260.94 | 64.60 | 170.04 | 146.07 |
| 183 | 6.78 | 100.58 | 104.79 | 197.19 | 15.45 | 317.49 | 167.56 |
| 184 | 2.77 | 118.38 | 29.80 | 134.87 | 3.66 | 249.75 | 123.32 |
| 185 | 9.39 | 183.69 | 29.99 | 196.64 | 24.55 | 299.07 | 167.83 |

TPP: thiamin pyrophosphate, TMP: thiamin monophosphate, FAD: flavin adenine dinucleotide
